# Mild Vacancy Generation and Acidification of the H–USY Zeolite with a Single-Electron Organic Donor

**DOI:** 10.1021/acsomega.5c02616

**Published:** 2025-06-04

**Authors:** Susi Hervàs−Arnandis, Marta Mon, Judit Oliver−Meseguer, Hermenegildo García, Antonio Leyva−Pérez

**Affiliations:** Instituto de Tecnología Química (Universitat Politècnica de València−Agencia Estatal Consejo Superior de Investigaciones Científicas), Avda. de los Naranjos s/n, 46022 València, Spain

## Abstract

The ultra-stabilized acidic Y (H–USY) zeolite is a robust, microporous, tridimensional, crystalline aluminosilicate widely employed as a solid catalyst in petrochemistry and fine chemistry, in virtue of its acidity and high internal surface area. Here we show that these two parameters, i.e., acidity and internal surface area, are enhanced when the H–USY zeolite is treated with a single-electron organic donor such as thianthrene, without any additional solvent nor additive. A spontaneous electron donation from thianthrene to the zeolite framework occurs, to generate the corresponding thianthrene cation radical and the negatively charged zeolite, which, counterintuitively, increases rather than decreases the zeolite acidity, after generating vacancies in the zeolite framework and increasing the surface area. The vacant H–USY solid (vac–H–USY) catalyzes the opening reaction of epoxides with alcohols and water with nearly twice faster reaction rates compared to the pristine H–USY, and the solid is recoverable and reusable. These results bring a mild and green methodology to generate vacancies in zeolites, circumventing current methodologies at >400 °C, and also to increase the surface area and acidity of the solid zeolite without altering neither its structure nor metal composition, with applications in catalysis and beyond.

## Graphical abstract

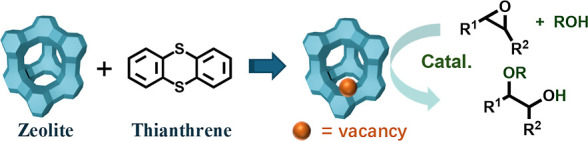

## 1 Introduction

Zeolites are the most used catalysts by volume worldwide.^1^ These microporous 3D aluminosilicates show narrow channels and cavities which direct the catalytic reaction toward the desired product with the help of selective adsorption properties and confined catalytic sites.^2^ Many of these reactions are acid-catalyzed, and a plethora of methods have been developed over the years to increase and design the acid sites within the zeolite framework.^2b^ For a given zeolite, a solid acid enhancement can generally be achieved by either increasing the framework Si/Al ratio, by doping the framework with more electropositive metals or by controlling the number and nature of the extra-framework counterbalancing cations.^3^ However, all these methods intrinsically involve a change in the chemical composition of the (semi)metal atoms inside the zeolite; thus, any method which may allow us to increase the acidity of the zeolite while keeping untouched its metal composition is of interest.

The generation of vacancies in the zeolite framework has been studied for years, since it was discovered early that the heating of the zeolite at >400 °C under an inert atmosphere or vacuum generates some vacancies in the framework, without significantly altering the crystalline structure.^4^ Figure 1A shows that these vacancies can be essentially of three types, in accordance with the chemical elements of the zeolite: O,^12^ Al,^7^ or Si^15^ vacancies. On one hand, O vacancies can be generated either by thermal^5^ or chemical treatments with powerful oxophilic metals, such as Mg(0).^6^ On the other hand, Al vacancies are generated after dealumination processes,^7^ usually with steam or other dealuminating reagents such as ammonium hexafluorosilicate.^8^ And, finally, Si vacancies are rarer and usually introduced on purpose with a post-synthetic reaction with SiCl_4_.^4^ In all cases, the processes can be expressed as two-electron reduction reactions, in order to generate the required vacancy.

**Figure 1:**
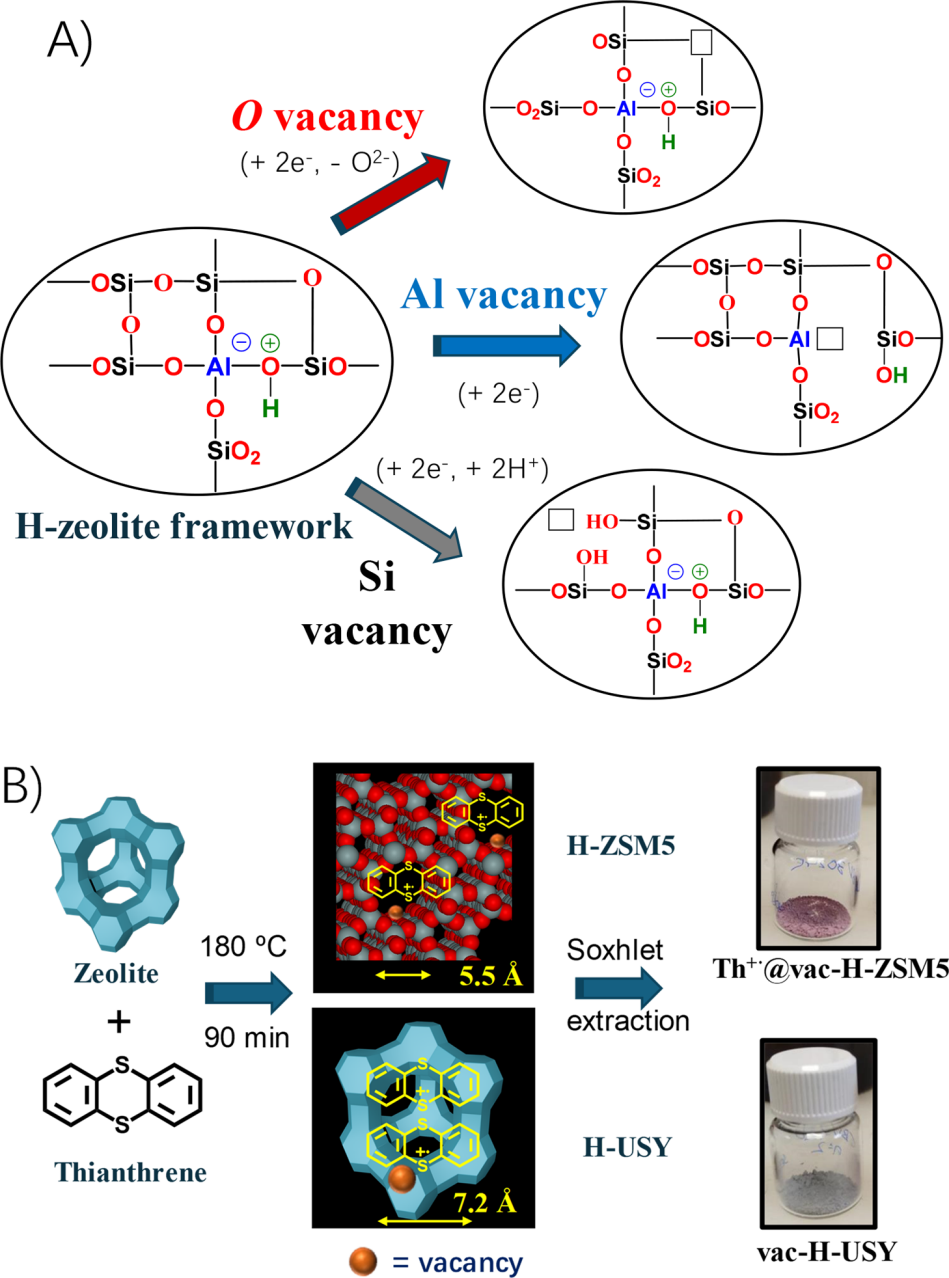
(A) Schematization of vacancy generation in a H–zeolite framework; the negative charges are compensated by extra-framework counterbalancing cations, in this case H^+^. (B) The synthetic approach here employed, with thianthrene as a one-electron radical donor.

The nature of the vacancy in a zeolite can be determined by a proper characterization of the material on the basis of the vacant atom fate.^9^ A combination of available techniques either supports or concomitantly discards each type of vacant generation.^10^ For instance, the vacancy of O is rapidly quenched in open air, leading to a loss of the vacant characteristics under ambient conditions. In contrast, the Al vacancies are more stable, and can be as uncoordinated framework Al sites (as represented in Figure 1A) or evolve to generate extra-framework Al species (EFAl), either interacting with the framework (framework associated Al species or FAAl) or not (Figure S1). For the latter, silanol nests may be created and further dehydrate to generate open Si atoms (Figure S1), which leads to an additional increase in the internal surface area in the zeolite. The generation of internal surface area is beneficial for catalytic purposes. It must be noted here that, currently, the relatively aggressive methods to obtain the different zeolite vacancies usually have as a collateral effect a diminution of the internal surface area of the solid, thus hampering the catalytic activity of the material. Finally, Si vacancies do not produce the described effects for Al but generate T-O sites, reflected in the ^29^Si NMR spectrum.

Thianthrene (Th) and related one-electron organic donors spontaneously oxidize in the presence of Brønsted acid zeolites, to give the corresponding cation radical and a reduced zeolite framework.^11^ Th is a cheap and widely available organic compound,^12^ which has been recently reported as an ubiquitous leaving group for coupling reactions.^13^ Figure 1B shows that the purple-colored thianthrenium cation radical (Th^+•^) persists indefinitely in the narrow channels (5.5 Å) of the pentasil H–ZSM5 zeolite, since the small dimension of the pores constrains and stabilizes the radical cation Th^+•^ (called here Th^+•^@vac–H–ZSM5), but in contrast, Th^+•^ dimerizes and neutralizes itself within the more open structure of the H–USY zeolite, a process allowed in the wider channels (7.7 Å) and in the supercavities (12.0 Å) of this faujasite 3D zeolite (called here vac–H–USY).^14^ In this way, the zeolite acts as a macromolecular anion, in line with recent reports on the isolation of Th^+•^ with certain anions.^15^

We show here that the electron donation of Th to H–ZSM5 and H–USY zeolites generates vacancies in the framework, which ultimately makes the final zeolite more acidic. However, in contrast to the H–ZSM5 zeolite, the final H–USY zeolite is not purple or white but gray, after self-quenching the Th^+•^ species. It has been proposed that this gray color is characteristic of atom vacancies in zeolites.^16^ We show here that a combination of techniques, including elemental analysis (EA); powder X-ray diffraction (PXRD); Brunauer, Emmett and Teller surface area (BET surface); diffuse reflectance adsorption and emission (fluorescence) ultraviolet–visible spectroscopy (DR UV–vis); ^27^Al and ^29^Si magic angle spinning solid-state nuclear magnetic resonance (MAS–SS–NMR); electronic paramagnetic resonance (EPR); X-ray photoelectron spectroscopy (XPS); high-resolution transmission electron microscope equipped with an energy-dispersive X-ray analyzer (HRTEM-EDX) and field emission scanning electron microscopy (FESEM); thermally controlled (temperature-programmed) Fourier-transformed infrared spectroscopy (TP–FT-IR) with or without amine probes; and NH_3_–temperature-programmed desorption (TPD), strongly supports the formation and dehydration of Al vacancies, to generate vacant Al sites and open O–Si–O sites in the H–USY zeolite framework. In this way, the more acidic vac–H–USY zeolite is able to catalyze two representative acid-catalyzed industrial reactions, i.e., the hydroalkoxylation and hydration of epoxides,^17,18^ at nearly twice faster reaction rates than the starting H–USY commercial zeolite. Despite zeolites having been reported as catalysts for the hydration reaction,^19^ it is difficult to find examples of zeolites as catalysts for the hydroalkoxylation of epoxides^20^ since the hydration reaction with the strongly adsorbed water or the epoxide rearrangement to aldehyde reaction tends to occur.^21^ Besides, the way of enhancing acidity and internal surface area shown here is, to our knowledge, unknown, and may compete with classical mesoporosity formation^22^ or additional metal atom incorporation strategies.^23^ The sterically crowded Th^+•^@vac–H–ZSM5 zeolite works with the smaller substrates (i.e., ethylene oxide and water), but it is the vac–H–USY catalysis which catalyzes the opening of a variety of epoxides with different alcohols and water.

## 2 Methods

### 2.1 General Synthesis of the Vacant Zeolites

The zeolite (1 g) was weighted in a 10 mL round-bottomed flask equipped with a stir bar. Thianthrene (80 mg) was added, and the mixture was placed in a magnetically stirred preheated oil bath at 180 °C. After 90 min, the mixture was cooled, and the solid was transferred to a paper cartridge and submitted to Soxhlet extraction with dichloromethane (DCM) for typically 6 h, until any coloration is not discharged from the solid. The resulting solid was dried in an oven at 100 °C.

### 2.2 Synthesis of Control H–USY zeolite (H–USY–Soxhlet)

The zeolite (1 g) was weighted in a 10 mL round-bottomed flask equipped with a stir bar and the mixture was placed in a magnetically stirred pre-heated oil bath at 180 °C. After 90 min, the mixture was cooled and the solid was transferred to a paper cartridge, and submitted to Soxhlet extraction with dichloromethane (DCM) for typically 6 h, until any coloration is not discharged from the solid. The resulting solid was dried on an oven at 100 °C.

### 2.3 Synthesis of Dealuminated Zeolite

The H–USY zeolite was treated with a 0.085 M solution of ammonium fluorosilicate for 20 h at room temperature to give the corresponding dealuminated HY zeolite. The resulting solid was filtered off and dried in an oven at 100 °C. Subsequently, the dealuminated HY zeolite was dried under vacuum at 250 °C overnight.

### 2.4 General Catalytic Reaction Procedure

The corresponding solid catalyst, the solvent/reactant (1 mL), and the corresponding epoxide were introduced in a 5 mL glass vial equipped with a magnetic stirrer. The vial was closed with a septum and placed in a magnetically stirred preheated oil bath at 60 °C for the required time. After the reaction was complete, in a filtration trough, a 25 mm nylon membrane filter was used to separate the solid catalyst. The reaction mixture was followed by taking 50 mL aliquots out, to be analyzed by GC and GC–MS, after diluting in an inert organic solvent (i.e., DCM) and adding *n*–octane or *n*–dodecane as an external standard.

## 3 Results and Discussion

### 3.1 Synthesis of the Vacant Acid Zeolites

The H–ZSM5, H–USY, and H–Beta zeolites employed here were obtained from commercial sources, while Al-MCM41 and ITQ-2 aluminosilicates were prepared according to known procedures. A list of the different physicochemical properties can be found in the Supporting Information (Table S1), together with some BET surface plots (Figure S2) and a representative field emission scanning electron microscopy (FESEM) image with the corresponding high-resolution transmission electron microscopy (HR-TEM) coupled to electron diffraction X-ray (EDX) analysis and mapping (Figure S3). These analyses confirm the given specifications of commercial sources. The procedure to prepare the vacant zeolites consists in heating 1 g of the corresponding dehydrated zeolite with typically 80 mg of Th (8 wt % with respect to the zeolite, 0.37 mmol·g^–1^) at 180 °C for 90 min, as shown in Figure 1B (see Table S2 for exact reactant amounts). The dehydrated zeolite was previously obtained after heating the solid at 250 °C under vacuum for 2 h, to remove ca. 40 mg of water (4 wt %), in the case of H–USY zeolite (SiO_2_/Al_2_O_3_ = 30 or Si/Al = 15). After the preparation reaction, the solid mixture was submitted to a Soxhlet extraction with dichloromethane (DCM) for 6 h in order to remove most of the organic thioaromatic compounds, except the ionically bounded radical cation Th^+.^ and some Th dimers occluded into the zeolite (Figure S4, right). In this way, a 0.82 wt % of Th (0.04 mmol·g^–1^) was incorporated for Th^+•^@vac–H–ZSM5 and a 1.40 wt % (0.06 mmol·g^–1^) for vac–H–USY (Table S3, entries 1–2). Thermogravimetric analysis (TGA) of vac–H–ZSM5 before and after Soxhlet washings was also performed (Figure S5). The results show that there is an extra weight loss of 3.4% between 124 and 235 °C for the Th^+•^@vac–H–ZSM5, which could be Th evaporation. These results are in agreement with the higher internal volume capacity and higher surface area of H–USY compared to H–ZSM5, in line with previous reports.^14^ The procedure can be scaled up to dozens of grams if desired (Figure S4, left). Please notice that the second step is just to remove the nonadsorbed material, but this step could be skipped for scaling up or kept to recover unused Th for further production.

The H–ZSM5 zeolite (SiO_2_/Al_2_O_3_ = 33) keeps the purple color corresponding to Th^+•^ after the Soxhlet extraction,^24^ while the H–USY zeolite with a similar Si/Al ratio (SiO_2_/Al_2_O_3_ = 30) turns gray (Figure S6, top). Other zeolites tested such as H–Beta, Al–MCM41, and the dealuminated ITQ-2 zeolite (keeping the same SiO_2_/Al_2_O_3_ = 30, or as approximate as possible) did not show any visible purple coloring during reaction or after Soxhlet extraction, but just brown coloring (Figure S6, bottom). It must be noticed that radical cations can be easily generated and stabilized in mesoporous structures such as that of Al-MCM41;^25^ thus the lack of formation of Th^+•^ in this and other porous aluminosilicates is related not only to the framework type but also to the presence of protons in the material. According to the latter, NaY zeolite (and also Na-Beta zeolite) did not show any coloring neither.

A longer time during the H–USY zeolite activation under vacuum (7 h instead of 2 h) led to a significant decrease in the final gray color of the vacant H–USY zeolite (Figure S6, top), which might be related to a higher loss of water (7 vs 4 wt %, see sample vac–H–USY–dehyd in Table S2, entry 3). This result is also in line with the reported need of protons to generate the radical cation Th^+•^,^14^ and suggests that the formation of radical cations and, in turn, the potential generation of vacancies in the zeolite, can be tuned as a function of the amount of water. The EA of the faujasite zeolites supports this assumption, since it shows that the amount of occluded Th species after the Soxhlet extraction is proportional to the amount of protons/water available (Table S3, entries 2–3).^26^ The efficiency of the Soxhlet extraction is also confirmed by the EA of the zeolite before and after extraction (Table S3, entry 4). These results are in line with the formation of dimeric species of Th into the H–USY zeolite, which is severely inhibited in the narrow channels of H–ZSM5 (Figure S6, bottom).^27^

The immersion of the Th-loaded zeolites in organic solvents and water does not produce any visual change in the solids, neither when immersed in the liquid nor when recovered after vacuum filtration and drying (Figure S7).^28^ The passage of steam through the Th^+•^@vac–H–ZSM5 zeolite did not produce any change in the strong purple color of the solid. Indeed, a sample of Th^+•^@vac–H–ZSM5 prepared in 2003, >20 years ago, still keeps the pink color (Figure S8).^29^ These results are representative of the robustness of the Th-loaded zeolitic material at ambient exposure and suggest that the modified zeolites could be used in aqueous reactive systems, for instance, as catalysts.

### 3.2 Characterization of the Vacant Acid Zeolites

The PXRD analysis of the solids after Soxhlet extraction shows that the crystalline structure of the starting H–ZSM5 and H–USY zeolites remains untouched after the treatment with Th (Figures S9–S11). In accordance, the corresponding FT-IR spectra show that the main Si–O and Al–O bands of the zeolite, before and after the Th treatment, remain unaltered (Figures S12 and S13). Besides, the main original signal at ≈1500 cm^–1^ in the FT-IR spectrum of the Th disappears in the zeolite materials, to give different aromatic signals at higher wavelengths (≈1600 cm^–1^), which supports the formation of the radical cations Th^+.^ and dimeric species.

Figure 2A shows the DR UV–vis spectra for Th^+•^@vac–H–ZSM5 and the corresponding experiment controls. The adsorption bands at 270, 290, and 540 nm, corresponding to the cation radical Th^+•^,^26^ can be clearly seen in the Th^+•^@vac–H–ZSM5 material, either treated or not with THF and H_2_O. This result is in good accordance with the visual inspection of the material after immersion in these solvents (see above), where the strong purple color is maintained. However, the nonextracted zeolite (before the Soxhlet extraction) mainly shows the band at 260 nm corresponding to unreacted Th, which is the major species in the unwashed solid with a ≈5:1 intensity with respect to the Th^+•^ (considering the corresponding absorptivity coefficients, ε), which correlates well with the amount of Th^+•^ loaded into the washed zeolite (see Table S3). Remarkably, in all the treated zeolites except the bare zeolite H–ZSM5, a wide but significant band between 290 and 330 nm appears, and this band is associated with vacancies in the zeolite framework. Moreover, we observed a broad band around 400–420 nm, indicative of silicon-associated hole centers. The former is more prominent in the vac–H–USY materials, as shown in Figure 2B. In contrast to H–ZSM5, the unreacted Th band does not appear with much intensity in the H–USY zeolite before the Soxhlet extraction, but mainly the Th^+•^ bands. However, these Th^+•^ bands are absent of the extracted zeolites, where the main bands correspond to Th^+•^ dimers and vacancies (regardless of zeolite immersion in THF or H_2_O). These results support the formation of vacancies in both Th–loaded H–ZSM5 and H–USY zeolites, and these vacancies are more abundant in the H–USY materials because more Th^+•^ species are formed and react within the wider microstructure of the 3D faujasite zeolite.

**Figure 2:**
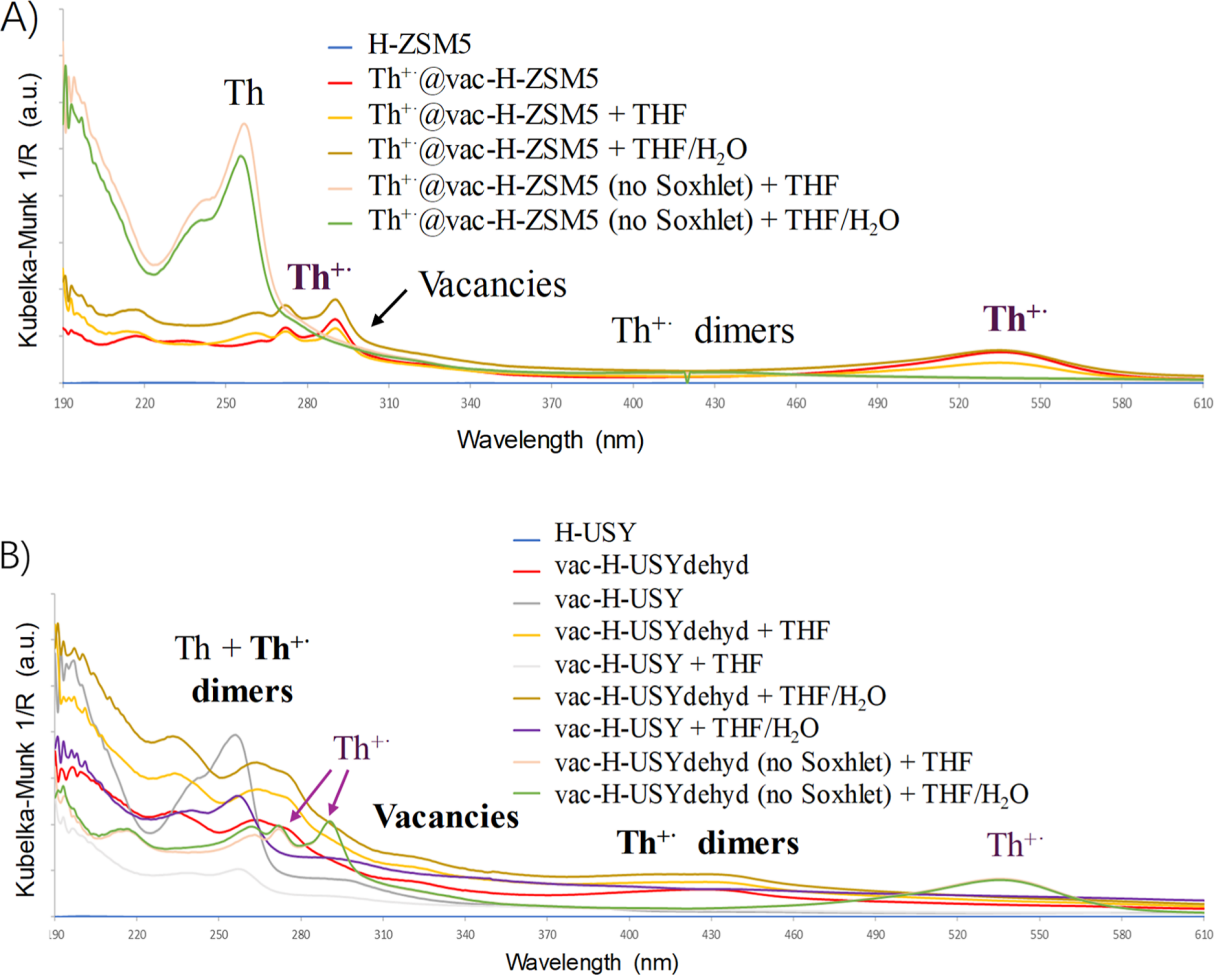
DR UV–vis spectra for (A) Th^+•^@vac–H–ZSM5 and B) vac–H–USY, with the corresponding starting materials and treatments in different solvents. The main diagnostic bands for Th, Th^+•^, dimers, and vacancies are indicated (in bold, the major species).

Figure 3 shows the fluorescence (UV–vis emission) spectra of the H–USY and vac–H–USY zeolites. Vacancies on zeolites are well-known to produce a large Stokes shift after irradiation,^16^ in contrast to the Th^+•^ species. The results show that any broad luminescence band does not appear when the H–USY zeolite is irradiated at 400–410 nm; however, a clear new band around 680–730 nm, with a maximum at 710 nm, appears after irradiating the vac–H–USY zeolite.

**Figure 3:**
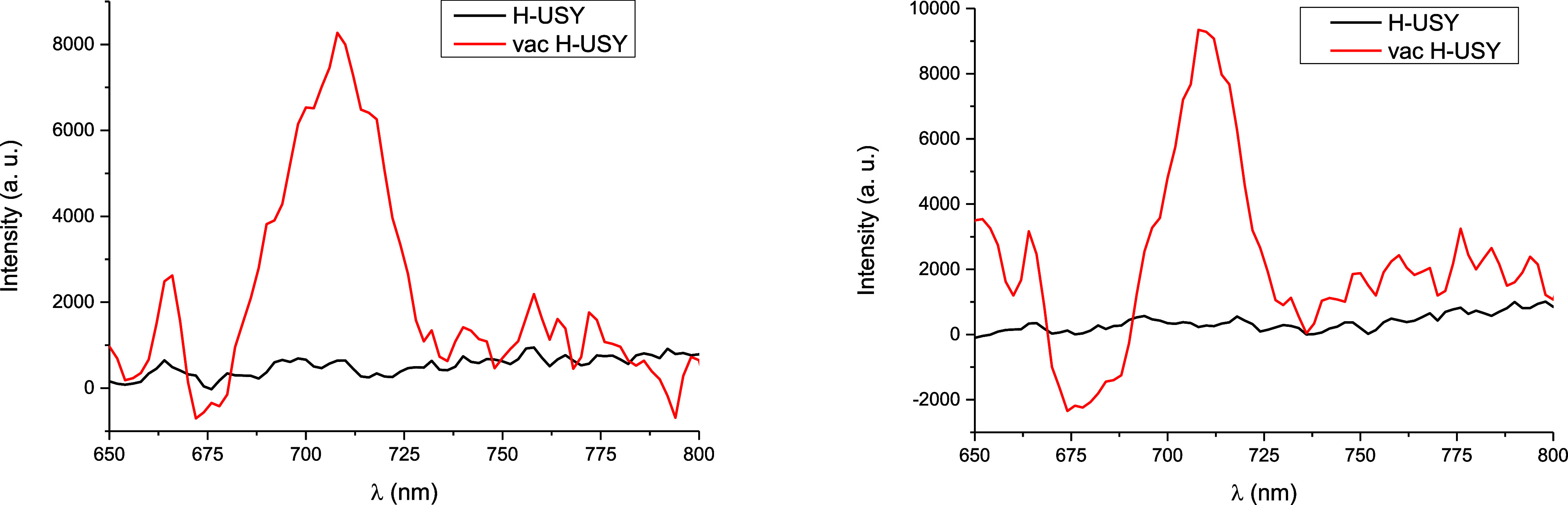
Left: Fluorescence emission spectra, using λ_exc_ = 400 nm at room temperature, for H–USY and vac–H–USY zeolites. Right: Fluorescence emission spectra, using λ_exc_= 410 nm at room temperature, for H–USY and vac–H–USY zeolites.

Besides, electronic paramagnetic resonance (EPR) measurements of the resulting vac–H–USY solid give the expected broad band with g= 2.004 for vacancies in the H–USY zeolite framework^16^ after treatment with Th (Figure S14), in contrast to the untreated zeolite or the supported but unreacted radical cation Th^+•^, which shows a well-defined and high intense signal with *g* = 2.009. These results strongly indicate formation of vacancies after reaction with Th.

Figure 4 shows the Si 2p_3/2_ XPS analysis of the bare H–USY sample after its treatment with Th. The results show that for the H–USY Si 2p region, two distinct peaks at 101.6 and 103.8 eV are detected, which can be attributed to the presence of Si atoms in SiO_*x*_ and aluminosilicates. In contrast, a new peak in the treated sample at 99.6 eV can be attributed to Si vacancies.^30^ The quantitative analysis shows that the amount of these Si vacancies fits well the ≈20% Th^+•^ generation and incorporation into the zeolite observed above. The corresponding O 1s XPS measurements confirm the formation of these Si–O–Si vacancies (Figure S15), and the relative area corresponding to nonbonding oxygen sites increased from H–USY (12.9%) to vac–H–USY (14.8%), indicating an increase in the concentration of defect sites. In other words, the XPS study provides a strong support for the generation of vacancies through the mechanism shown in Figure 1B, which consists of the formation of vacant Al sites in the framework and, probably, with dehydration to give new Si open sites (see Figure S1). Besides, the absence of S signals in XPS confirmed the low amount of Th that remained inside the zeolite after forming the vacancies. The generation of vacancies can also be associated with a dealumination plus dehydration reaction, to give EFAl sites that, in any case, are not octahedral Al species. The Al 2p_3/2_ XPS analysis shows the disappearance of the octahedral Al sites during the treatment with Th, in accordance with the generation of vacancies (Figure S16).

**Figure 4:**
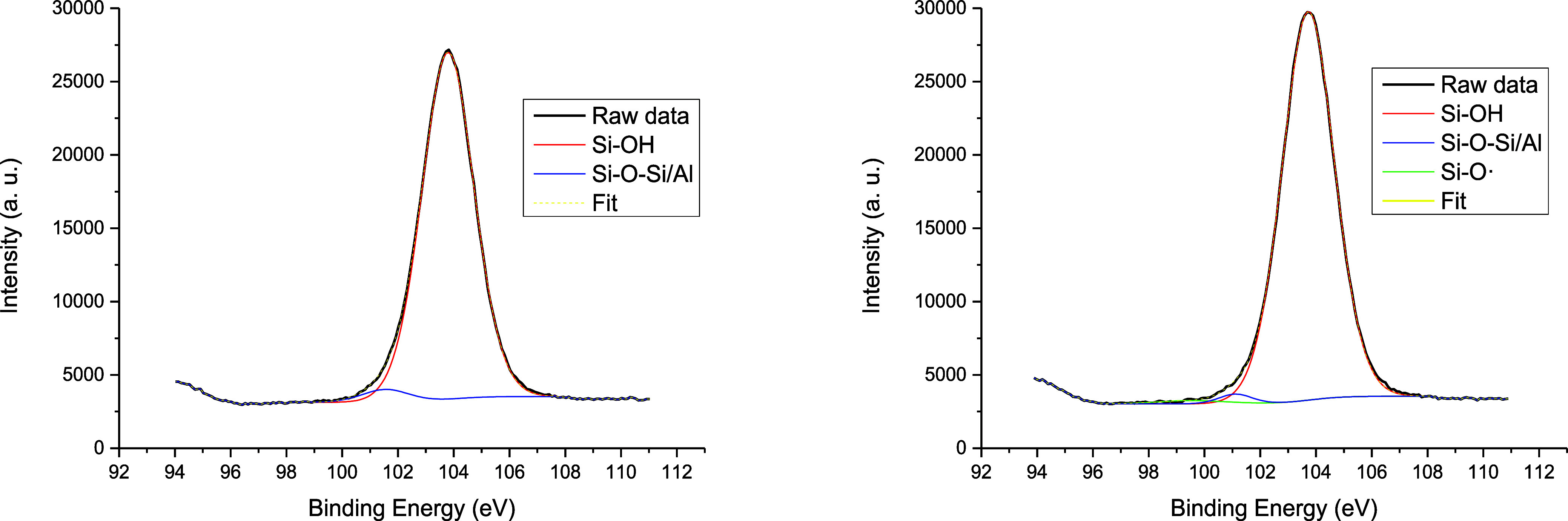
Si 2p XPS spectra for H–USY (left) and vac–H–USY (right) zeolites. The green line corresponds to the vacancies.

^27^Al MAS–SS–NMR measurements were carried out in order to confirm the formation of the zeolitic Al extraframework after the treatment with Th. It must be said here that these experiments require a previous thermal activation of the zeolite (>300 °C under vacuum) in order to remove as much water as possible, since the latter can interfere during the experiments; thus the Th^+•^@vac–H–ZSM5 can be unstable under these relatively harsh dehydration conditions. Figure 5 shows the spectra of vac–H–USY compared to those of the pristine H–USY zeolite, and it can be clearly seen that any formation of additional octahedral Al sites does not occur after reaction with Th. If this is so, the amount of silanol nests should have not been increased yet after the Th treatment, since it constitutes the first step during the dealumination process. The corresponding ^29^Si MAS–SS–NMR measurements did not show any increase in the amount of silanol nests, but an increase in the Q4 sites ([Si–(OSi)_4_] bonds), thus confirming the lack of severe dealumination processes and the possible formation of new T–O sites (open O_3_–Al sites and new Si–O–Si bonds, Figure S17). FT-IR measurements of the dehydrated zeolites at 150 °C confirm that the final vac–H–USY material does not present a significant amount of any new silanol (Figure S18). These results strongly support the idea that vacancy generation is accompanied by a rapid dehydration process of the so-generated silanols to give new Si–O–Si bonds (see Figure S1). The Th^+•^@vac–H–ZSM5 sample was anyway studied by ^27^Al MAS–SS–NMR and the spectrum (Figure S19) shows the expected tetrahedral Al sites together with some additional pentahedral and octahedral Al sites, which could indicate the generation of some EFAl sites either during the treatment with Th or the thermal treatment.

**Figure 5:**
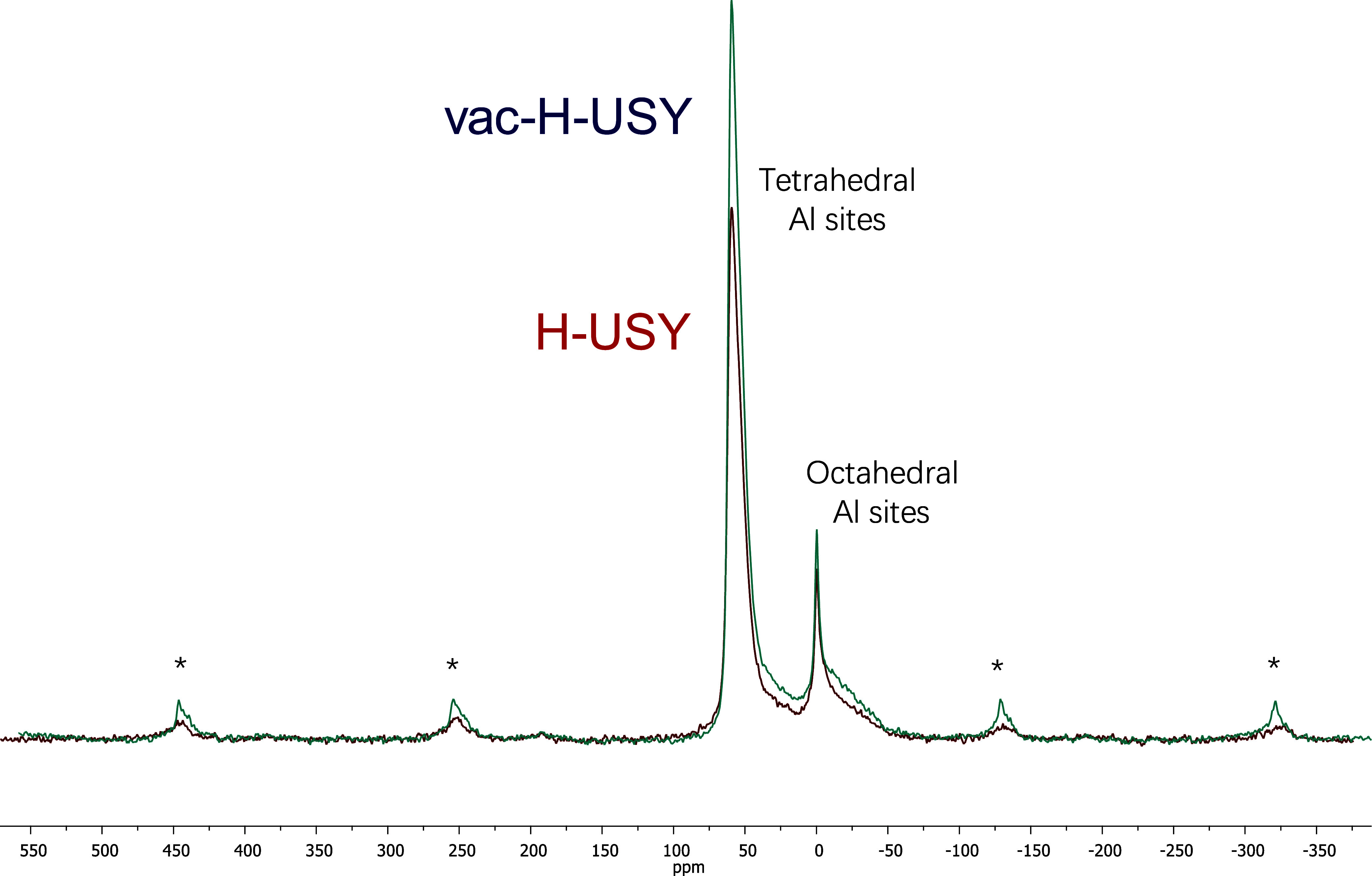
^27^Al MAS–SS–NMR spectra for vac–H–USY (blue line) and H–USY (red line). The spectrum for vac–H–USY is a little magnified to clearly compare both lines. Asterisks (*) denote spinning signals.

Powder X-ray diffraction (PXRD) for the Th^+•^@vac–H–ZSM5 samples before and after Soxhlet washing was also performed (Figure S20). We observe that a characteristic peak from the HZSM5 zeolite structure, at 12.5°, disappears. The intensities of the peaks at lower θ are the same, suggesting that the Th is not inserted in the channels of the zeolite. The stability of the crystalline structure was confirmed by high-resolution transmission electron microscopy (HR-TEM), and the dispersion of thianthrene within the zeolite structure was demonstrated by energy-dispersive X-ray (EDX) spectroscopy. The presence of sulfur atoms throughout the crystal structures of both H–USY (Figure S21) and H–ZSM-5 (Figure S22) confirms the incorporation of Th into both zeolites.

As commented on above, the formation of these new Si–O–Si bonds should be accompanied by a slight but significant increase in the internal (microporous) surface area of the zeolite. To check this, BET surface area measurements of the Th-treated zeolite vac–H–USY were performed, and the result (Figure S23) shows an increase in the surface area from 593 in the starting H–USY zeolite to 758 m^2^·g^–1^ for the vac–H–USY zeolite (Table S1), with an increase in the micropore volume from 0.44 to 0.52 cm^3^·g^–1^. Indeed, this increase in surface area occurs exclusively in the microporous internal area, which increases from 502 to 682 m^2^·g^–1^ in vac–H–USY, an increase of ≈25% with respect to the starting zeolite. These results are remarkable considering that the Th species are still occluded in the zeolite, thus the real increase in the internal zeolite surface has to be higher. This result contrasts with and improves some of the main methods to prepare vacancies in aluminosilicates, based on aggressive reagents such as Mg metal of high calcination temperatures, where the surface area of the material decreases rather than increases.^16^ Here, the occurrence of the dealumination/dehydration mechanism after treatment of the zeolite with the one-electron organic donor, depicted in Figure 1B, enables a mild generation of vacancies with concomitant increase of surface area and acidity (see next section).

### 3.3 The Nature of the Acidity in the Vacant Zeolites

The formation of vacant Al and Si sites in the active zeolites Th^+•^@vac–H–ZSM5 and vac–H–USY should be translated in the formation of additional Lewis acid sites.^3^ To check this, NH_3_–TPD experiments were performed for the H–USY and vac–H–USY zeolites (as it occurred above for the EPR and BET experiments, Th^+•^@vac–H–ZSM5 could not be studied due to the potential decomposition under thermal dehydration conditions). The comparative results show that 22% more NH_3_ is adsorbed in the vac–H–USY zeolite (10.18 cm^3^·g^–1^) than in the starting H–USY zeolite (8.32 cm^3^·g^–1^), and that more and stronger acid sites are present in the vac–H–USY zeolite according to the desorption temperature curves (Figure S24). Thermogravimetric mass analysis during the TPD experiments confirms these results (Figure S25). In order to assess the nature of these additional acid sites in the vac–H–USY zeolite, TP–FT-IR experiments with pyridine as a probe were performed, and Figure 6 shows the results at a 150 °C desorption temperature. A clear increase in the amount of Lewis acid sites can be seen and not in the amount of Brønsted sites, and this increase fits well with the ≈20% of more acid sites encountered by the ^27^Al MAS–SS–NMR and NH_3_–TPD techniques. Additional TP–FT-IR experiments also reveal that these acid sites are stable up to 350 °C and that further dealumination does not occur at that high temperature, since bridging hydroxyls are barely generated (Figure S26). Thus, one can say that the treatment of the H–USY zeolite has provoked an increase of ≈20% in the Al Lewis sites, together with an increase of ≈25% of the internal surface area, without any damage in the robustness and integrity of the zeolitic framework.

**Figure 6:**
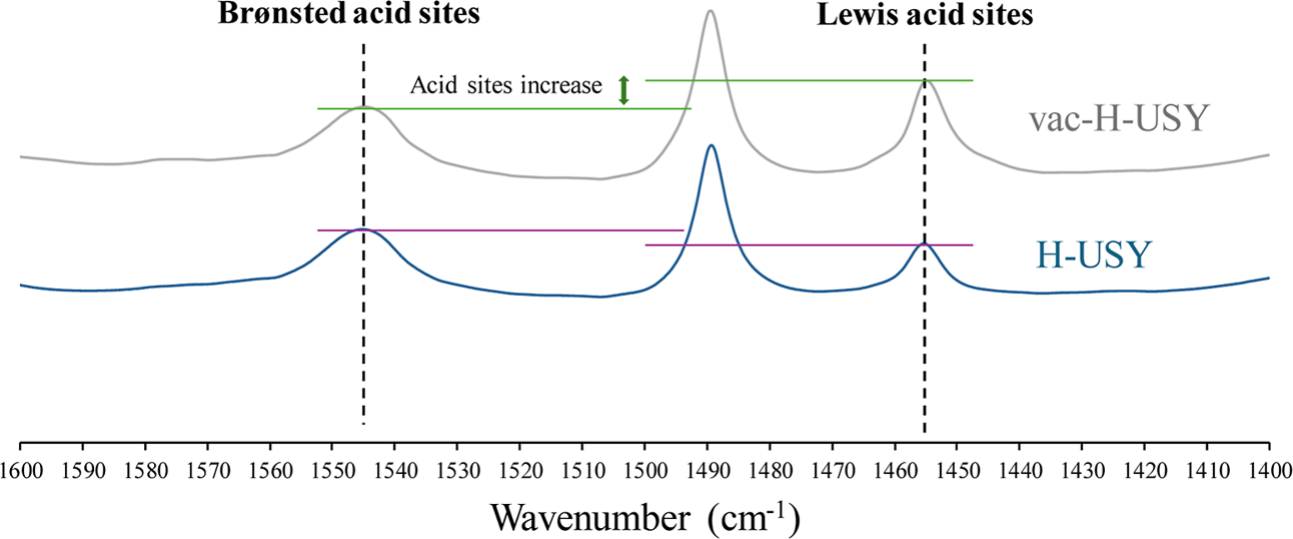
Pyridine-probe TP–FT-IR spectra of the diagnostic area for vac–H–USY (top, gray line) and H–USY (bottom, blue line) after thermal activation at 150 °C under vacuum, indicating the corresponding Brønsted and Lewis sites. Straight lines are a guide to the eye.

The integration of the signals in Figure 6 gives an amount of generated Lewis sites in vac–H–USY ≈0.1 mmol·g^–1^, after using the extinction molar coefficients given by Emeis.^31^ This number doubles the amount of Th incorporated in the zeolite, in this case 0.06 mmol·g^–1^ (see Table S3). Notice that the zeolite framework acts as a whole, delocalizing the received electrons until the vacancies are generated and the framework becomes electroneutral.^32^ This result also explains the observed increase in Th^+•^ formation with the Al content of the zeolite.^33^

### 3.4 Catalytic Epoxide Opening

The combined increase in surface area and acidity in the Th-loaded zeolites, particularly in vac–H–USY, brings the possibility of using them as solid catalysts for acid-catalyzed reactions. Here, we tested the opening of epoxides with alcohols and water, which, as said above, is an industrial reaction currently catalyzed by soluble and unrecoverable strong acids (such as BF_3_·OEt_2_ or H_3_PO_4_),^17^ thus of much interest to be performed with mild and recoverable solid acids.^18^ It is difficult to find any precedent on the hydroalkoxylation of epoxides with zeolite catalysts,^20^ in contrast to the hydration^19,22,23^ and aminolysis reactions,^20,34^ despite related reactions such as the methoxylation of alkenes being well studied with zeolites.^35^ For that reason, we focused first on the methoxylation reaction of two different epoxides, and Figure 7A shows the kinetic results for the hydroalkoxylation reaction of *p*–Cl–styryl epoxide **1a** in MeOH solution (1 M). The kinetic profiles were calculated after following the reaction by gas chromatography (GC) in the presence of either of the zeolites at the same weight (5 wt % respect to the limiting reactant **1a**), and it can be seen that the vac–H–USY zeolite shows the faster initial rate, 1.25 times higher than vac–H–USY–dehyd and 1.53 times higher than H–USY. Moreover, we also compared the zeolite treated after Soxhlet extraction, without the addition of Th, and the lowest initial rate and final yield was obtained for the latter (H–USY–Soxhlet). Additionally, we also compared with another vacant zeolite, such as dealuminated-H–USY zeolite prepared following a previous reported procedure,^8^ with Al vacancies in the framework, confirming that these vacancies are not responsible for the increased activity (Figure S27).

**Figure 7:**
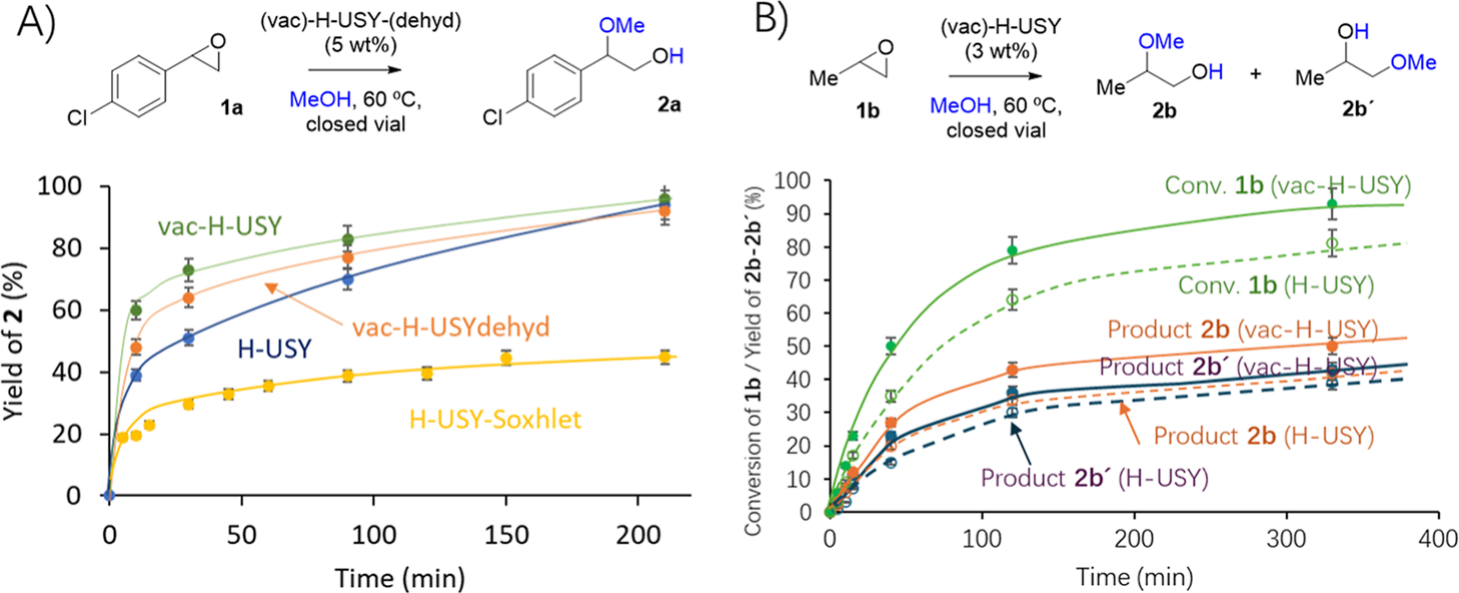
Kinetic plots for the hydroalkoxylation reaction of (A) *p*–Cl–styryl epoxide **1a** or propylene epoxide **1b** (B) with MeOH (1 M) in the presence of H–USY, vac–H–USY, vac–H–USY–dehyd zeolite, and H–USY–Soxhlet (3 or 5 wt %) at 60 °C, to give products **2a**, **2b**, and **2b́**, respectively. No oligomeric products were found. GC results. Lines are a guide to the eye (solid and dashed lines correspond to the Markovnikov and anti-Markovnikov products, respectively). Error bars account for a 5% uncertainty.

According to the pyridine TP–FT-IR values, the number of acid sites per vac–H–USY is 0.7 mmol·g^–1^, which means that 2.5 μmol (0.25 mol %) of acid sites are under operation, which gives a considerable initial turnover frequency (TOF_0_ = 1440 h^–1^) in solution (the pyridine acidity values were taken here instead of the NH_3_–TPD values in order to discard the inaccessible acid sites). The Markovnikov product **2a** is exclusively encountered, as assessed by ^1^H and ^13^C NMR after performing the reaction in CD_3_OD and stopping at 25% conversion (Figure S28), due to the strong activating effect of the aromatic ring on the α-carbon atom. Any common oligomeric^36^ or rearrangement to aldehyde^21^ byproducts were not observed. The steric effect imparted by the occluded Th species in the vac–H–USY zeolite catalyst is not detrimental here since, indeed, the TOF_0_ is higher than for the smaller propylene epoxide **1b** substrate (see ahead). Indeed, kinetic tests at different stirring speeds do not show any variation in the initial reaction rate for *p*–Cl–styryl epoxide **1a** (Figure S29), which indicates that mass diffusion within the zeolite is not rate-controlling yet.^37^ In contrast, the Th^+•^ species in Th^+•^@vac–H–ZSM5 provokes a very strong steric hindrance for epoxide **1a**, to give just a minor catalytic activity (4%·h^–1^ for Th^+•^@vac–H–ZSM5 vs 28%·h^–1^ for H–ZSM5, and 360%·h^–1^ for vac–H–USY), in line with the difficult diffusion of **1a** within the occupied narrow channels of H–ZSM5.

A second epoxide methoxylation reaction, in this case that of propylene epoxide **1b**, was carried out, and the results are shown in Figure 7B. The kinetic profiles in the presence of either H–USY or vac–H–USY zeolite (3 wt %) clearly indicate that the vac–H–USY zeolite is more active than the H–USY zeolite, specifically 1.6 times in the initial rate (84%·h^–1^ vs 54%·h^–1^). The TOF_0_ calculated is, in this case, 840 h^–1^, which is lower than for epoxide **1a**. The vac–H–USY–dehyd zeolite, with a lower amount of Th loaded into the solid, gave again an intermediate catalytic behavior between H–USY and vac–H–USY (1.2 times the initial rate of H–USY, not shown for the sake of clarity), in accordance with a slightly higher acidity than H–USY.

The selectivity to products **2b** and **2b′** was quite similar to all the zeolites, with a 10% improvement for the Markovnikov (more substituted) addition product **2b**, as assessed by GC coupled to mass spectrometry (GC–MS) and ^1^H NMR of the reaction with CD_3_OD as a solvent. Remarkably, further addition reactions (i.e., addition of products **2b** and **2b′** to epoxide **1b**, to form oligomers) were not encountered either by GC–MS or by NMR, which may be related to the steric limitations imposed by the zeolite. The reaction could be scaled up to 30 mmol of **1b** to give the same high yield (97%) and selectivity for products **2b**:**2b’** (60:40) after 5 h of reaction time at 60 °C, to obtain ≈2 g of products after filtration of the solid catalyst and controlled removal of the volatiles under vacuum (Figure S30). In contrast, the Th^+•^@vac–H–ZSM5 zeolite could only give a 42% conversion of propylene epoxide **1b** under the same reaction conditions (though with a similar product selectivity), a lower yield than any H–USY zeolite and even lower than the pristine H–ZSM5 zeolite (61% yield and 4 times higher initial reaction rate than Th^+•^@vac–H–ZSM5, 24%·h^–1^ vs 5%·h^–1^), showcasing that the excessive steric hindrance provoked by the Th^+•^ units severely hampers any catalytic activity. It is noteworthy to say that the selectivity to products **2b–2b′** of bare H–USY and H–ZSM5 is not distinct (see Figure 7B) but the catalytic activity is much lower for the epoxide opening reaction; thus, the loading of Th species makes the difference.

These results are in line with the higher Lewis acidity and surface area of the vac–H–USY zeolite, which enhances the catalytic activity of the solid for the acid-catalyzed epoxide opening reaction of epoxides while keeping a complete selectivity for the monoaddition products **1b** or **2b–2b′**. In fact, this reaction has been reported to mostly occur on zeolitic Lewis acid sites,^23,38^ which explains that an increase of ≈20% Lewis sites in the zeolite provokes such catalytic enhancement.

Figure 8 illustrates the scope of the epoxide opening reaction using 12 different epoxides and 3 different alcohols [methanol (products **2**), ethanol (products **4**) and benzyl alcohol (products **5**)], and water (products **3**). The results indicate that all of the epoxides can be successfully opened, exhibiting high selectivity in most cases. The stronger the nucleophile, the higher the yield is, thus the order of reactivity is MeOH > EtOH > H_2_O ≈ BnOH. For the latter, the steric hindrance imposed by the zeolite pores might play a role, which is also extensible for the epoxides. The obtained products include aromatic (**2a–4a**, **2c–5c** and **3l**), open chain aliphatic (**2b–3b**, **2f**, **4f**, **2g**, **4g** and **2h**), cyclic aliphatic (**2d–5d**, **2i–k**, **4e**, and **4j**), and molecules with oxygen-sensitive functional groups (**2h** and **2i**). Notably, a key result was the opening of limonene oxide with methanol or ethanol, where the major product was the Markovnikov product (**2d**) or the anti-Markovnikov product (**5d́**), respectively. This result might be related to the zeolitees’ steric hindrance, which also explains the low yields for large epoxides even with H_2_O (product **3l**).

**Figure 8:**
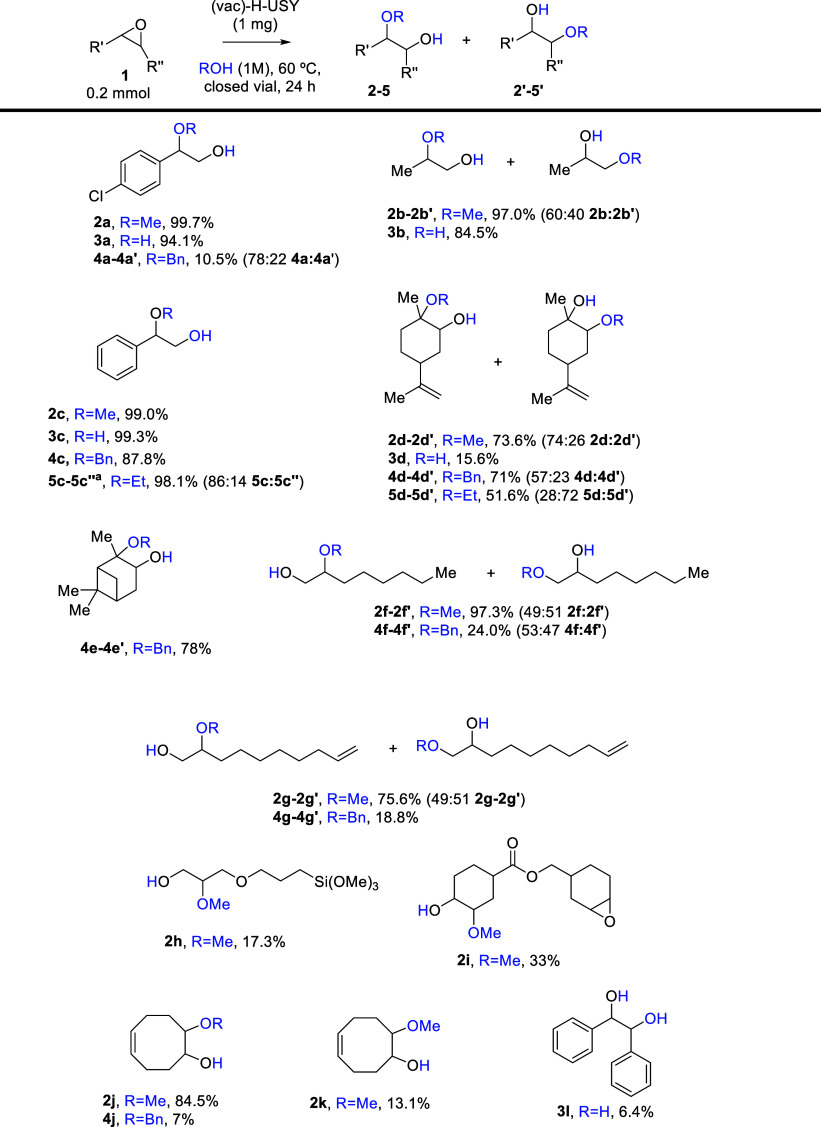
Scope of the epoxide opening with H_2_O and different alcohols. Conversions and selectivity are reported as GC results with *n*–dodecane as an internal standard. ^a^ The ethyl product also corresponds to the addition of 2 ethanol fragments in the epoxide, with a 14% selectivity.

### 3.5 Leaching and Reuses

A hot filtration test was carried out to assess the presence or not of catalytically active species in solution during the methoxylation reaction of *p*–Cl–styryl epoxide **1a** in MeOH (1 M) at 60 °C, with the vac–H–USY zeolite (2 wt %) as a catalyst. The kinetic results in Figure 9A show that after filtration of the solid catalyst, the reaction is completely stopped. Notice that the reaction was performed with a smaller amount of vac–H–USY zeolite than under optimized reaction conditions, to slow down the initial rate and filter off the solid at the appropriate conversion (≈20%). This result confirms that the zeolite acts as a truly heterogeneous catalyst, and in accordance with this, Figure 9B shows that the solid could be completely recovered by centrifugation and reused at least 10 times, after washings, without any depletion in the yield of product **2a**. Moreover, we were also able to reuse the catalyst during 10 times after using water as a reactant, to open the epoxide and maintain the final conversion. The PXRD analysis of the catalysts after 10 uses with MeOH shows that the crystalline structure of the functionalized zeolite is maintained after use, confirming the robustness of the new material prepared (Figure S31). The vac–H–USY zeolite was also reused 4 times with EtOH as the nucleophile, and the PXRD analysis also shows the robustness of the crystal structure (Figure S31).

**Figure 9:**
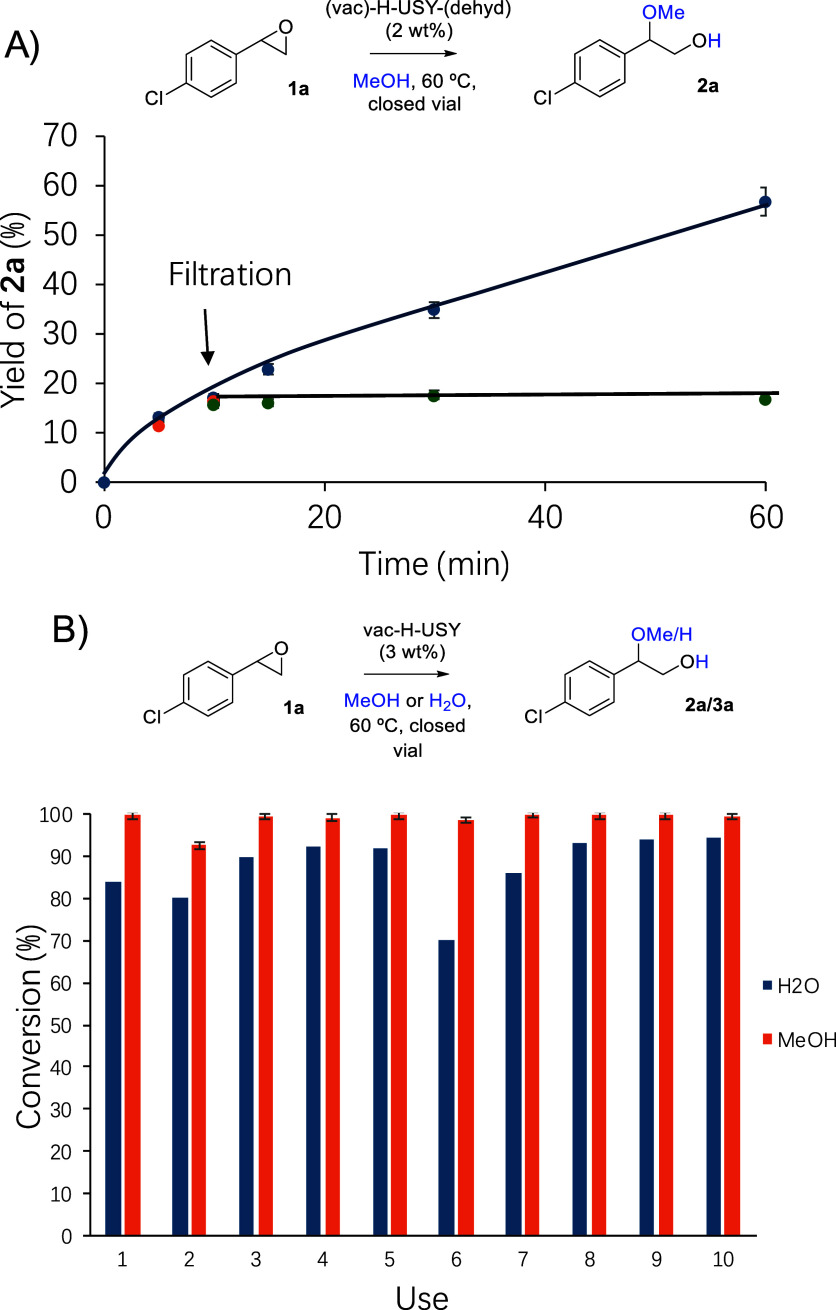
(A) Leaching test for the hydroalkoxylation reaction of *p*–Cl–styryl epoxide **1a** with MeOH (1 M) in the presence of vac–H–USY zeolite (2 wt %) at 60 °C, to give product **2a**. The filtration point is indicated. Blue, orange, and green points correspond to the solid-catalyzed reaction and a twin reaction before after filtering off the solid, respectively. (B) Results for the reuses of the vac–H–USY zeolite (2 wt %) in water and in methanol. Error bars account for a 5% uncertainty.

A new hot filtration test, in this case for the methoxylation reaction of propylene epoxide **1b**, shows some leaching during the catalyzed reaction, calculated to be 42% after filtration of the zeolite at 50% conversion (Figure S32). The selectivity of the reaction to products **2b** and **2b′** does not change significantly after removal of the solid. The presence of some catalytic active species in solution when using epoxide **1b** as the starting material is probably related to the small size and high polarity of the reagents, which may provoke the Al extraframework leaching out in the very polar protic hot medium. However, these species may re-incorporate onto the solid after cooling the mixture at the end of the reaction and, indeed, a 40% catalytic activity is recovered in a second use after re-activation of the zeolite at 250 °C under vacuum. A thermal re-activation is required to remove strongly adsorbed species, which include not only the remaining epoxide **1b** and methanol but also the very polar products **2b** and **2b′**, in accordance with the estimated rate equation (see ahead). Nevertheless, the methoxylation of **1b** could be carried out in the gas phase, thereby avoiding any leaching.^39^

### 3.6 Mechanism of the Catalytic Epoxide Opening

The reaction rate order for each reactant was estimated after varying the corresponding concentrations and applying the pseudostationary approximation through initial rate measurements for both epoxides **1a** and **1b**, with MeOH as a nucleophile at 60 °C reaction temperature (Figures S28 and S29). According to the kinetic profiles obtained, the following rate equation rates can be proposed: *v*_0_ = *k*_exp**1a**_[cat]­[MeOH]­[**1a**]^−1^ and *v*_0_ = *k*_exp**1b**_[cat] [**1b**]^−1^. The linear correlation between amount of catalyst and initial reaction rate is not surprising and confirms the good diffusion of the reactants within the microporous solid catalyst because any poisoning effect is not observed at lower catalytic amounts.^40^ In contrast, the amount of MeOH only appears in the equation for the bigger epoxide **1a**, and the kinetic isotopic effect (KIE), also calculated by kinetic experiments after using the initial rates to compare the relative reactivity of MeOH and CD_3_OD, gives a KIE_**1a**_ = 1.6(0) for epoxide **1a** and KIE_**1b**_ = 0.8(8) for epoxide **1b**, which matches the reaction orders found in the rate equations.

The results above indicate that the breaking of the O–H bond in MeOH intervenes in the rate-determining step (rds) of the alkoxylation reaction of **1a,** but not of **1b**. The epoxide concentration decreases linearly the initial reaction rate at 0.2–0.5 M concentration and even exponentially at higher concentrations (see Figures S33 and S34), which indicate a strong coordination of the epoxides to the acid catalytic site, regardless of the size. These results suggest that the coordination and activation of the epoxide onto the catalytic acid sites of the zeolite is behind the rds of the reaction,^36^ and Figure 10 depicts the mechanism proposed.

**Figure 10:**
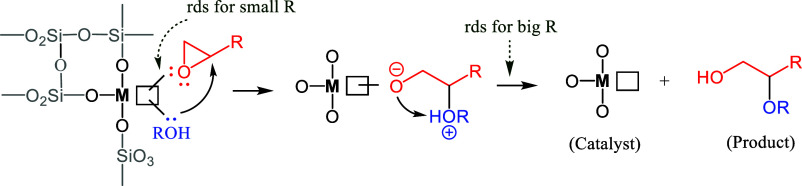
Proposed mechanism for the vacant zeolite-catalyzed hydroalkoxylation reaction of epoxides. M = Al or Si, rds: rate-determining step (small R refers to, for example, methyl; big R refers to, for example, phenyl).

## 4 Conclusions

Vacant H–ZSM5 and H–USY zeolites can be prepared by thermal treatment (180 °C, 90 min) with an organic single-electron donor such as thianthrene, without any other additive nor solvent required. After removing the excess of thianthrene, the zeolite achieves a 25% higher internal surface area and a 20% more Lewis acidity, in virtue of the generation of Al and Si vacancies in the solid framework, without affecting the overall stability of the zeolite. Combined elemental and acidity measurements, together with a complete characterization of the vacant material, show that vacancies are generated by the electrons injected with the organic donor to the framework, in accordance with a 2-electron reduction process for the (semi)metal vacancy generation. The vacant H–USY zeolite shows an enhanced catalytic activity (>1.5 times) with respect to the commercial starting zeolite for representative acid-catalyzed industrial reactions in the liquid phase, i.e., the hydroalkoxylation and hydration of epoxides. The vacant solid can be recovered and reused 10 times without depletion of the catalytic activity. The reaction mechanism shows the active role of the Lewis acid sites to activate the epoxide in the presence of the nucleophilic alcohol or water. The results here bring a mild procedure to generate vacancies in commercial acid zeolites for catalysis and, perhaps, other applications.^41^

## Supplementary Material



## Notes

Additional experimental data and compound characterization (PDF)
