# Supplementary material for: Mild Vacancy Generation and Acidification of the H–USY Zeolite with a Single-Electron Organic Donor
Source: ACS Omega. 2025 Jun 4;10(23):25014–26. doi: 10.1021/acsomega.5c02616 (PMC12177782; doi:10.1021/acsomega.5c02616)
Supplement: Supplementary file 1 [file ao5c02616_si_001.pdf]

SUPPORTING INFORMATION

Mild vacancy generation and acidification of the H–USY zeolite with a single-electron organic donor

Susi Hervàs-Arnandis, Marta Mon, Judit Oliver-Meseguer, Hermenegildo García and Antonio Leyva–Pérez.*

Instituto de Tecnología Química. Universidad Politècnica de València–Agencia Estatal Consejo Superior de Investigaciones Científicas. Avda. de los Naranjos s/n, 46022, Valencia, Spain.

Corresponding author: anleyva@itq.upv.es

Index

Experimental Section. 3

- General. 3

- Experimental techniques. 3

- Experimental procedures. 5

Figures S1-S34. 7

Tables S1-S3. 41

Compound characterization. 44

Experimental Section.

- General.

Glassware was dried in an oven at 175 ºC before use. Reactions were performed in 5 to 100 ml round-bottomed flasks equipped with a magnetic stirrer and closed with a rubber septum part to sample out if necessary. Reagents and solvents were obtained from commercial sources and were used without further purification otherwise indicated. Products were characterised by GC–MS, ^{1}H– and ^{13}C–NMR, and DEPT, and compared with commercial products if possible or the given literature.

- Experimental techniques.

Gas chromatographic analyses were performed in an instrument (Agilent 8860) equipped with a 30 m x 250 µm x 0.25 µm Agilent HP-50+ capillary column. *N*–dodecane was used as an external standard. GC–MS analyses were performed on an Agilent 8890N spectrometer equipped with a 30 m x 250 µm x 0.25 µm Agilent HP-5MS UI capillary column and operated under the same conditions. Products were characterized by comparison with the given literature, when possible.

^{1}H, ^{13}C, and DEPT nuclear magnetic resonance measurements were recorded in a 300 MHz or 400 MHz instrument using $CDCl_3$ or CD_3OD as a solvent, containing TMS as an internal standard.

Inductively coupled plasma-optical emission spectroscopy (ICP-OES) for atomic content was determined after disaggregating the solids in aqueous HF and dilution before analysis.

X-ray diffractograms (XRD) were recorded in a CubiX PRO (PAN Analytical) spectrometer, with a Cu K(α) radiation source, 1.5406 Å wavelength.

Solid FT-IR spectra of the compounds were recorded on a Jasko 460 plus spectrophotometer by impregnating the windows with a dispersion of the solid in a volatile organic solvent and leaving it to evaporate before analysis, or pressurization on a Ge disk.

N_2 adsorption-desorption isotherms were performed at 77 K on sieved zeolites after outgassing for 16 h under vacuum.

Field emission scanning electron microscopy (FESEM) were carried out after supporting the zeolite on a grid and measuring with a ZEISS Ultra-55 instrument, from Oxford Instruments.

High resolution transmission electron microscopy (HR–TEM) coupled to Energy-dispersive X-ray (EDX) spectroscopy measurements were performed in a 200 KeV Jeol JEM–2100 microscope by impregnating a copper–carbon filmed grid with a drop of the corresponding solid after dispersing in dichloromethane and leaving evaporation for at least 15 h.

Diffuse reflectance UV–Visible spectrophotometry (DR UV–vis) in the region comprised between 190 and 1100 nm was recorded at room temperature on a spectrophotometer equipped with an integrating sphere. The mixture was contained in a quartz cell with 1 mm path length; the layer can therefore be regarded as infinitely thick, as required by the Kubelka–Munk theory. Absorbance values (Al) were calculated from reflectance ones (Rl) according to the Kubelka–Munk transformation: Al = (1–Rl) 2/2Rl.

Emission spectra were obtained on a FLS1000 spectrometer (Edinburgh Instruments) equipped with a 400 W xenon lamp, double grating Czerny-Turner monochromators with 2 × 325 mm focal length in excitation and detection, and a PMT-980 detector in a cooled housing which covers a range from 200 to 980 nm.

Solid-state nuclear magnetic resonance spectra were recorded at room temperature with a Bruker AVIII HD 400 WB spectrometer. The ^{27}Al spectra were recorded with $\pi/12$ pulse length of 1 μs, and a recycle delay of 3 s, pinning the samples at 20 kHz.

FTIR-pyridine studies were performed in a Nicolet Is-10 Thermo FT-infrared spectrophotometer with self-supported pellets, degassed under vacuum at 300 ºC for 12 hours, followed by the introduction of pyridine into the cell at 650 Pa. After equilibrium was attained, the cell was degassed at a desired experiment temperature and cooled down to room temperature. We then acquired FTIR at 150 ºC (weak strength), 250 ºC (medium strength), and 350 ºC (high strength). A spectrum was collected under vacuum before pyridine adsorption, to be used as a background. We subtracted the background from each spectrum and normalized the absorbance to weight before calculations.

Temperature-programmed Fourier transform infrared (FT-IR) spectra were recorded on a Bruker Vertex 70 spectrometer equipped with a DTGS detector. The experiments were

carried out in a quartz IR cell allowing in situ treatments in controlled atmospheres and connected to a vacuum system with gas dosing capacity. Prior to the adsorption, the sample was evacuated at 200 ºC under vacuum (10^{-6} mbar) for 1.5 h.

X-ray photoelectron spectroscopy (XPS) measurements were performed after sticking the solid material onto a molybdenum plate with scotch tape fil, using a SPECS spectrometer equipped with a Phoibos 150 MCD-9 analyzer using non-monochromatic Mg KR (1253.6 eV) X-ray source working at 50 W. As an internal reference for the peak positions in the XPS spectra, the C1s peak has been set at 284.5 eV.

Electronic paramagnetic resonance (EPR) measurements were performed at 100K using an EMX–10/12 Bruker spectrometer working at the X band, with a frequency modulation of 100 kHz and 1G amplitude. The different zeolites were dried under vacuum and then introduced inside an EPR quartz probe cell to be measured.

- Experimental procedures.

General synthesis of the vacant zeolites.

The zeolite (1 g) was weighted in a 10 mL round-bottomed flask equipped with a stir bar. Thianthrene (80 mg) was added, and the mixture was placed in a magnetically stirred pre-heated oil bath at 180 ºC. After 90 min, the mixture was cooled and the solid was transferred to a paper cartridge, and submitted to Soxhlet extraction with dichloromethane (DCM) for typically 6 h, until any coloration is not discharged from the solid. The resulting solid was dried on an oven at 100 °C.

Synthesis of control H-USY zeolite (H-USY-Soxhlet).

The zeolite (1 g) was weighted in a 10 mL round-bottomed flask equipped with a stir bar and the mixture was placed in a magnetically stirred pre-heated oil bath at 180 ºC. After 90 min, the mixture was cooled and the solid was transferred to a paper cartridge, and submitted to Soxhlet extraction with dichloromethane (DCM) for typically 6 h, until any coloration is not discharged from the solid. The resulting solid was dried on an oven at 100 °C.

Synthesis of dealuminated zeolite.

The H-USY zeolite was treated with a 0.085 M solution of ammonium fluorosilicate, for 20 h at room temperature, to give the corresponding dealuminated HY zeolite. The

resulting solid was filtered off and dried on an oven at 100 °C. Subsequently, the dealuminated HY zeolite was dried under vacuum at 250 °C overnight.

General catalytic reaction procedure.

The corresponding solid catalyst, the solvent / reactant (1 mL) and the corresponding epoxide were introduced in a 5 mL glass vial equipped with a magnetic stirrer. The vial was closed with a septum and placed in a magnetically stirred pre-heated oil bath at 60 ºC, for the required time. After the reaction is complete, filtration trough a 25 mm nylon™ membrane filter is carried out, to separate the solid catalyst. The reaction mixture was followed by taking 50 µL aliquots out, to be analyzed by GC and GC-MS, after diluting in an inert organic solvent (i.e. DCM) and adding *n*-octane or *n*-dodecane as an external standard.

Leaching test.

Two identical reactions were prepared following the procedure described above. For each reaction, samples were taken at the same time and, after typically 10 min (20-50% conversion for epoxides **1a** and **1b**, respectively), one of the reactions was filtered using a PTFE hydrophobic 0.22µm syringe filter and left to react for an additional time to determine if any change in the reactant conversion can be observed (GC monitoring).

Reusability tests.

In a 1 mL glass vial equipped with a magnetic stirrer, 2.5 mg of vacant zeolite, 0.5 mmol of **1a**, and water or methanol as solvent / reactant (0.5 mL) were introduced. The vial was closed with a septum and placed in a magnetically stirred pre-heated oil bath at 60 ºC, for 24 h. After this time, the reaction mixture was centrifugated and the supernatant was analyzed by GC after diluting in DCM, adding *n*-dodecane as an external standard. The solid was washed with DCM, centrifugated and dried under vacuum to add again the epoxide and the solvent in the same vial for the next zeolite use.

Figures.

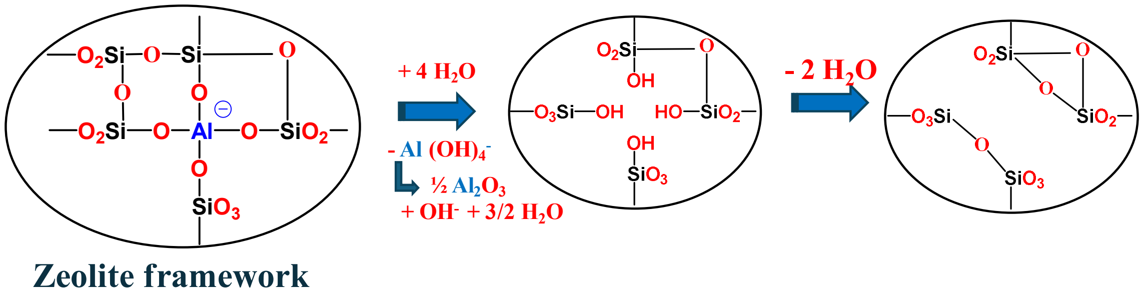

Figure S1. Schematization of the generation of silanol nests, EFAl or FAAl after dealumination / dehydration processes. The counteraction is not represented in this case.

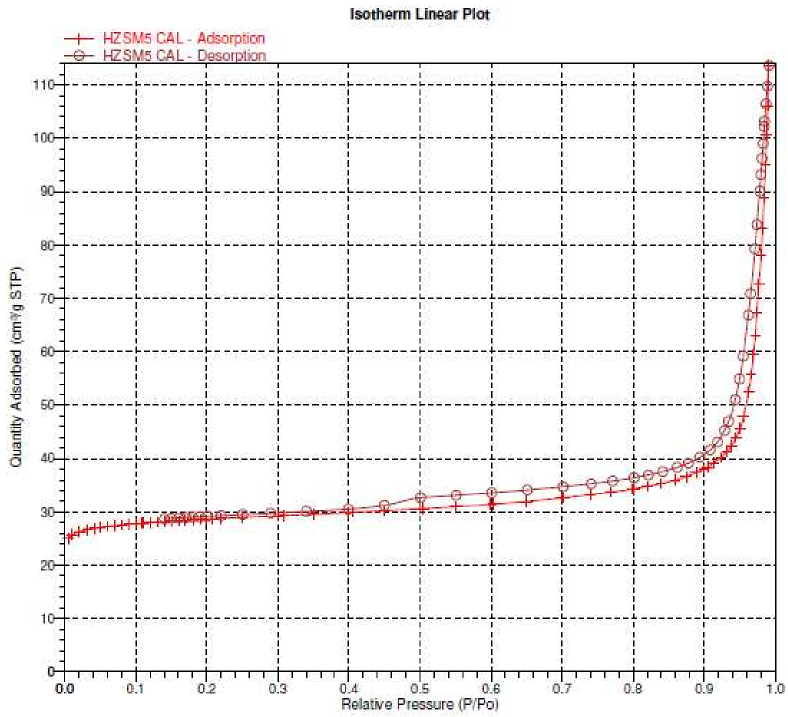

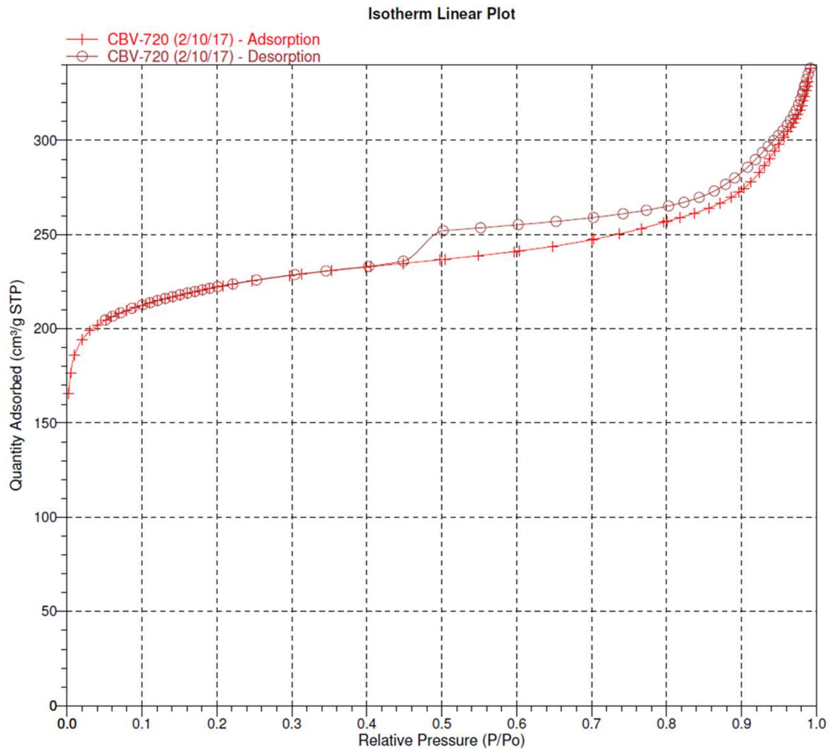

Figure S2. Isotherm plots for the H-ZMS5 zeolite after calcination (top) and the H-USY zeolite (bottom). For the latter, the zeolite sample name corresponds to the commercial notation.

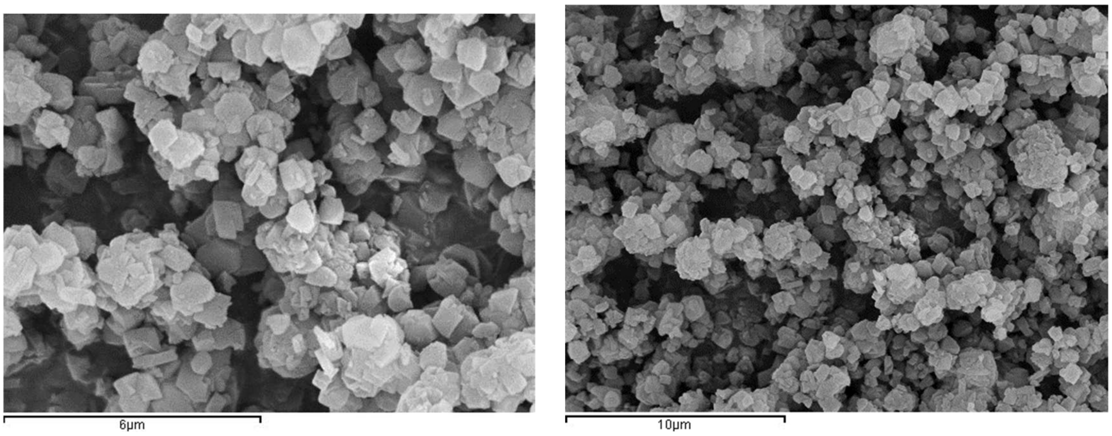

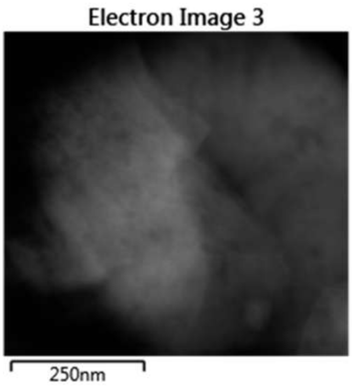

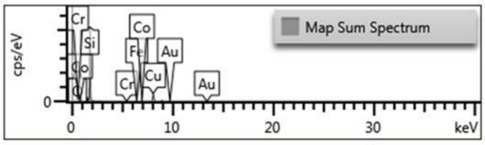

Element	Line Type	k Factor	k Factor type	Absorption Correction	Wt%	Wt% Sigma
C	K series	2.769	Theoretical	1	0.25	0.03
O	K series	2.02	Theoretical	1	36.06	0.12
Al	K series	1.049	Theoretical	1	2.19	0.04
Si	K series	1	Theoretical	1	47.68	0.12
Cr	K series	1.114	Theoretical	1	0.1	0.01
Fe	K series	1.143	Theoretical	1	0.09	0.01
Co	K series	1.185	Theoretical	1	0.12	0.02
Cu	K series	1.247	Theoretical	1	12.57	0.07
Au	L series	2.287	Theoretical	1	0.95	0.06
Total:					100	

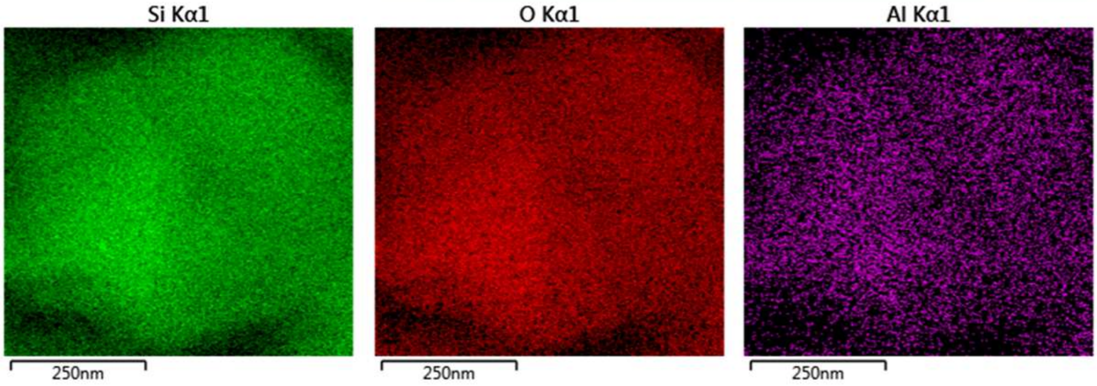

Figure S3. Representative field emission scanning electron microscope (FESEM) images of H-USY zeolite (top) and the corresponding high-resolution transmission electron microscopy (HR-TEM) images, with the electron diffraction X-ray (EDX) analysis and mapping.

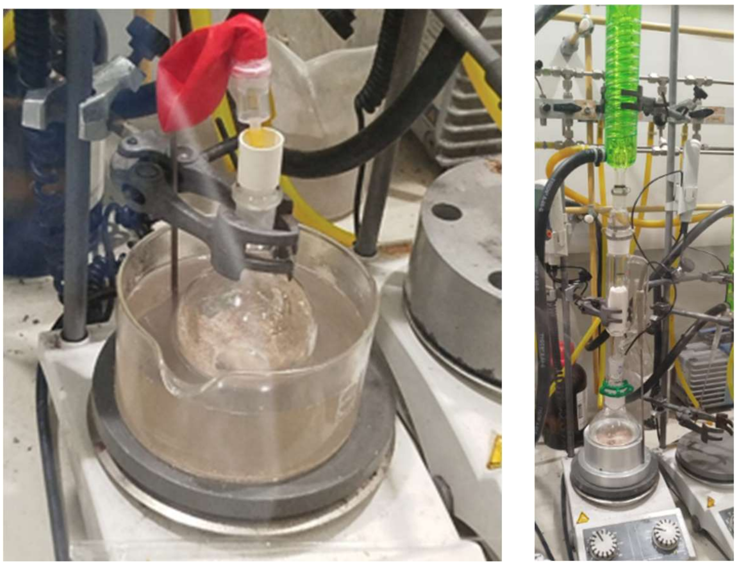

Figure S4. Left: Set-up of the multi-gram zeolite-thianthrene reaction. A punctured balloon is used to prevent the entry of ambient humidity and control the pressure due to possible gases generated during the process. Right: Set-up of the Soxhlet extraction. The solid sample is introduced into a well-pressed paper cartridge, through which the extraction solvent (DCM) circulates, evaporating and condensing, after setting the oil bath at 60 ºC for 6 h.

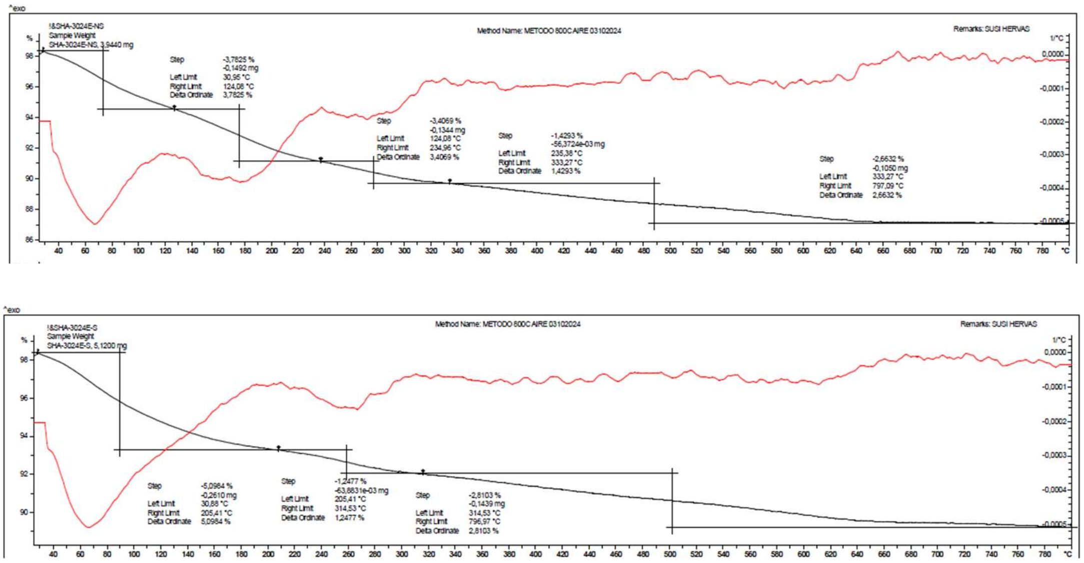

Figure S5. Thermogravimetric analysis (TGA) of vac-HZSM-5 before (top) and after Soxhlet washing (bottom). The loss of mass below 100 ºC corresponds to adsorbed water or organic volatiles. The red line shows a differential scanning calorimetry (DSC).

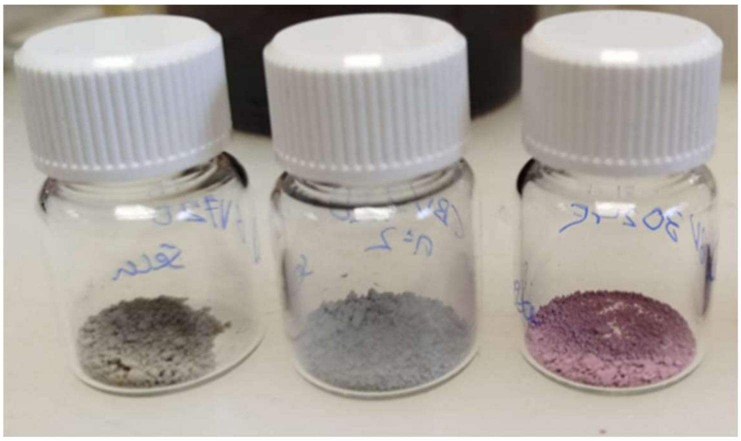

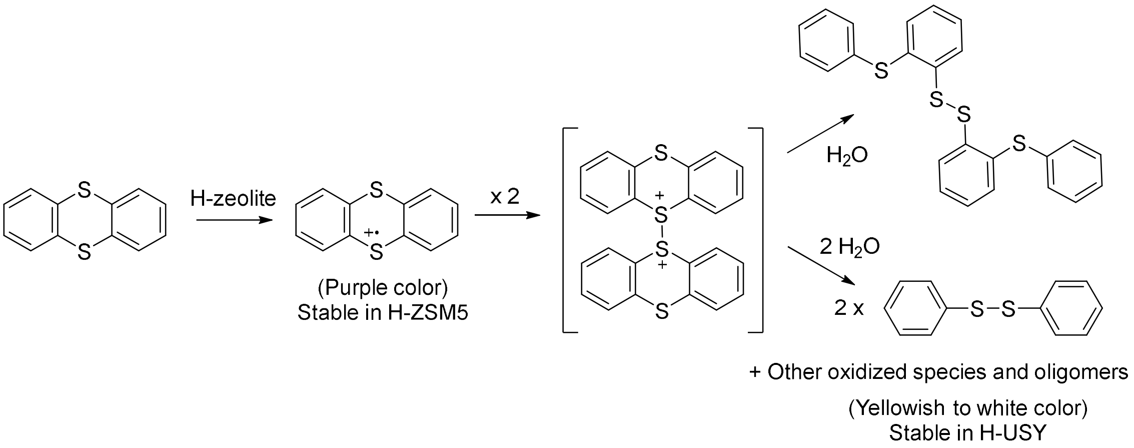

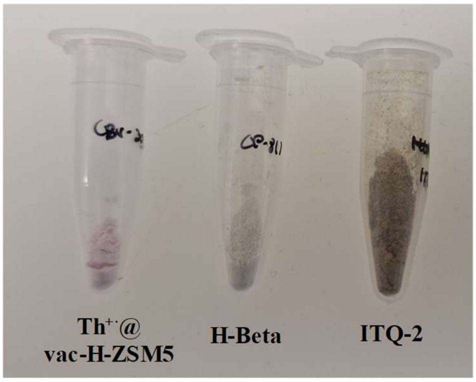

Figure S6. Top: Appearance of the active zeolites after the insertion of thianthrene Th, from left to right: vac-H-USY-dehyd, vac-H-USY and $Th^{+\cdot}$@vac-H-ZSM5; the formation of the thioaromatic species within the protic zeolites is shown below. Bottom: Final appearance of some of the non-active zeolites during the reaction with Th, where the pink color was not observed.

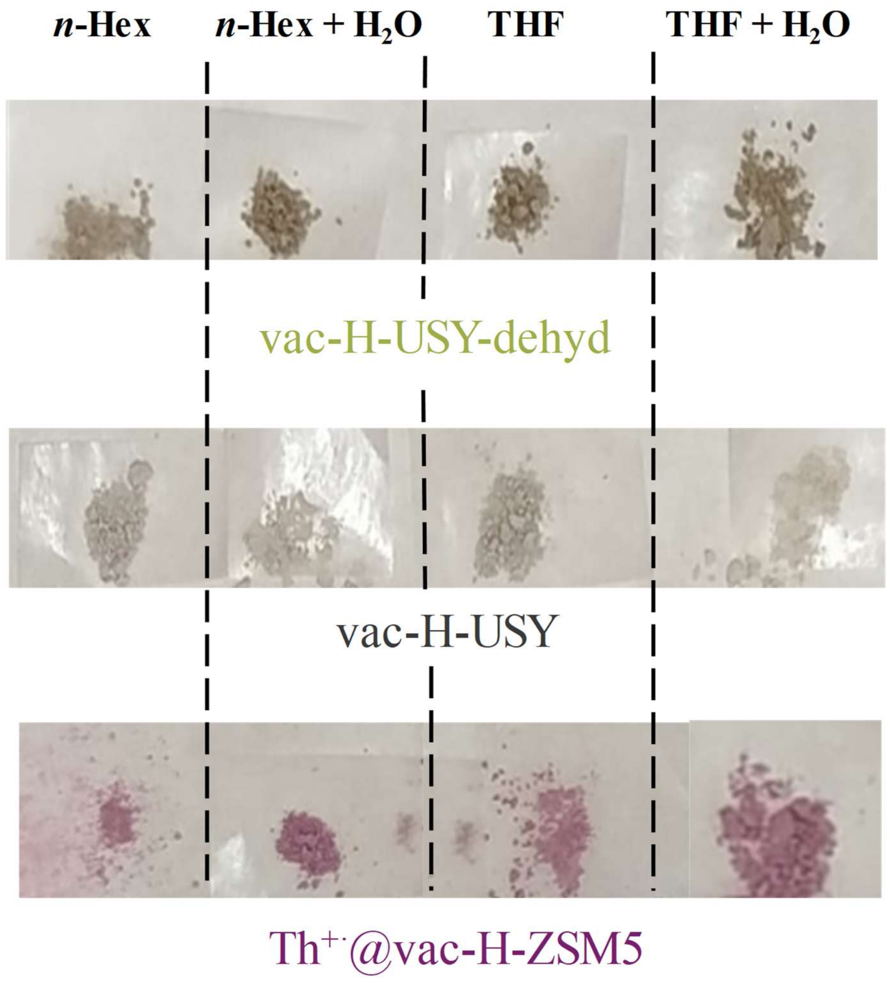

Figure S7. Appearance of the active zeolites after immersion at room temperature in the indicated solvents, vacuum filtration and drying at the open air. Top: vac-H-USY-dehyd, middle: vac-H-USY, bottom: $Th^{+\cdot}$@vac-H-ZSM5.

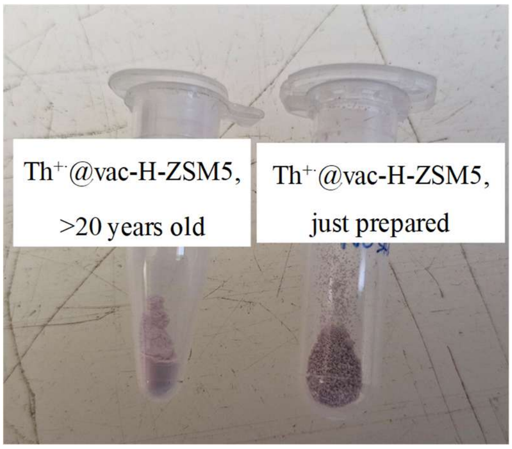

Figure S8. Photograph of a sample of $Th^{+\cdot}$@vac-H-ZSM5 prepared in May 2003 (left) and a fresh prepared sample (right).

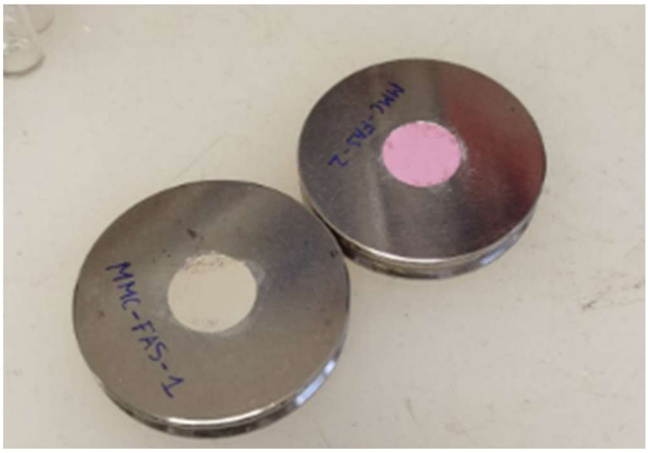

Figure S9. Samples to be analyzed by PXRD, from left to right: vac-H-USY-dehyd and $Th^{+\cdot}$@vac-H-ZSM5.

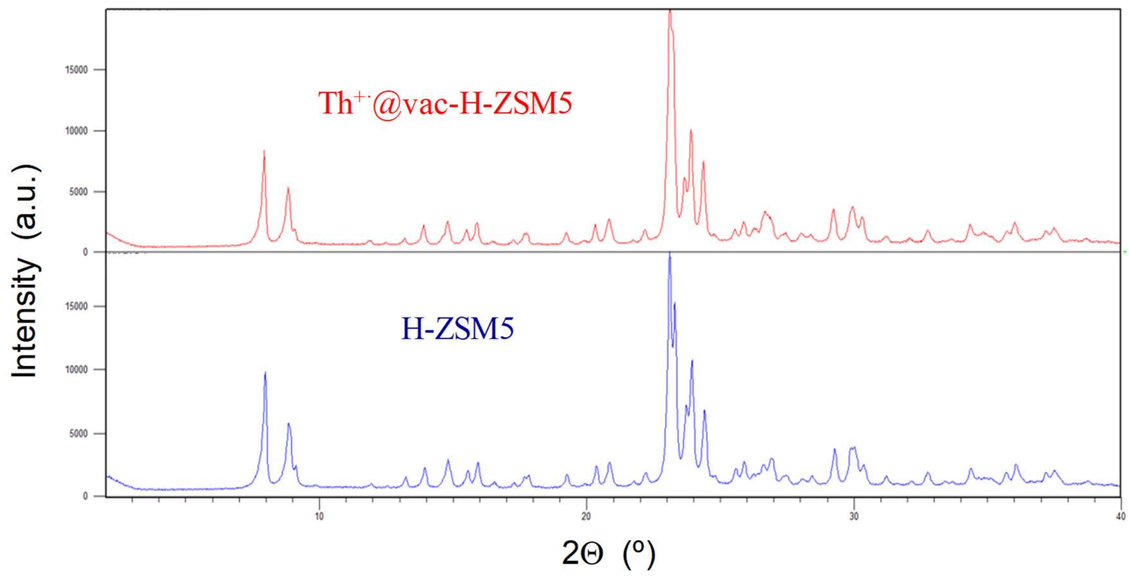

Figure S10. PXRD of $Th^{+\cdot}$@vac-H-ZSM5 (top) and H-ZSM5 (bottom).

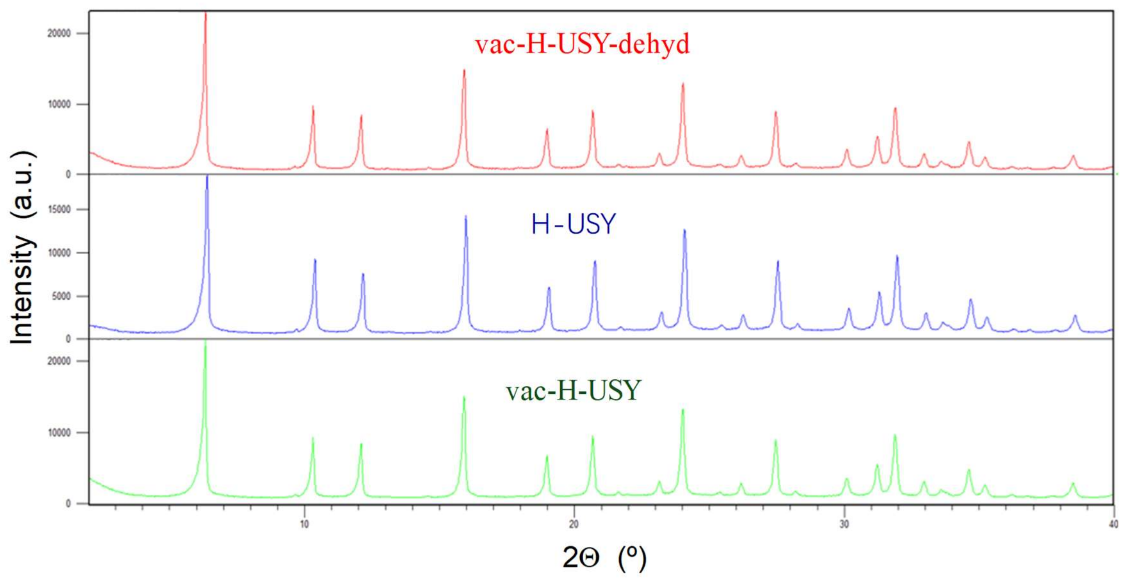

Figure S11. PXRD of vac-H-USY-dehyd (top), H-USY (middle) and vac-H-USY (bottom).

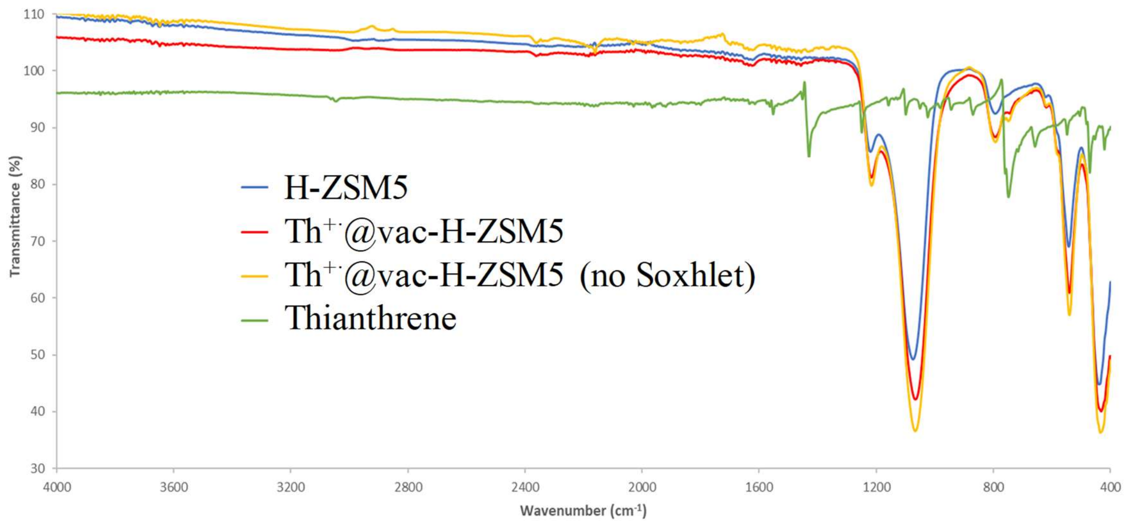

Figure S12. FT-IR of H-ZSM5 (blue line), $Th^{+\cdot}$@vac-H-ZSM5 after (red line) or before Soxhlet extraction (yellow line), and thianthrene Th (green line).

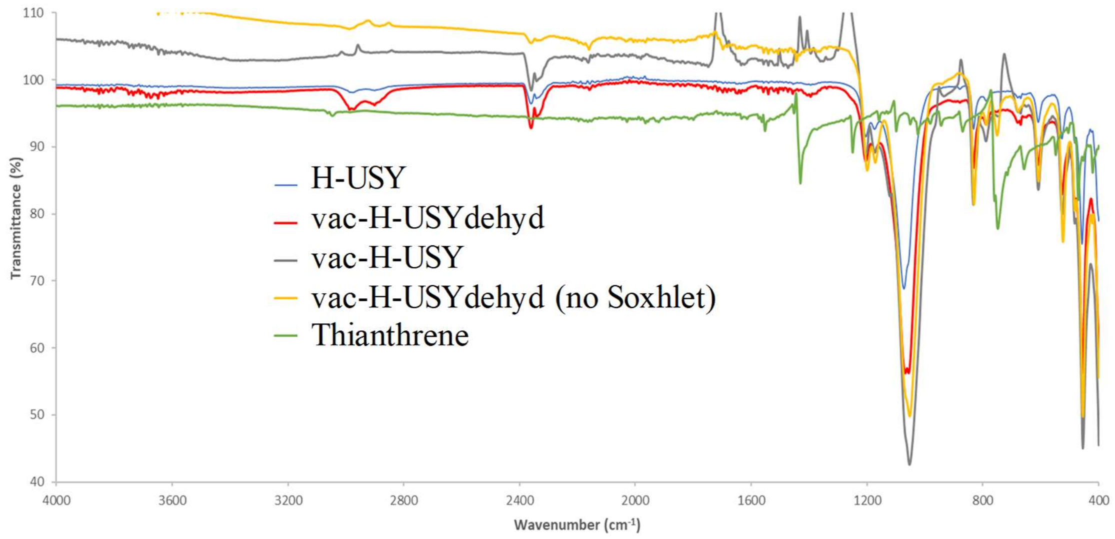

Figure S13. FT-IR of H-USY (blue line), vac-H-USYdehyd after (red line) or before Soxhlet extraction (yellow line), vac-H-USY (red line), and thianthrene Th (green line).

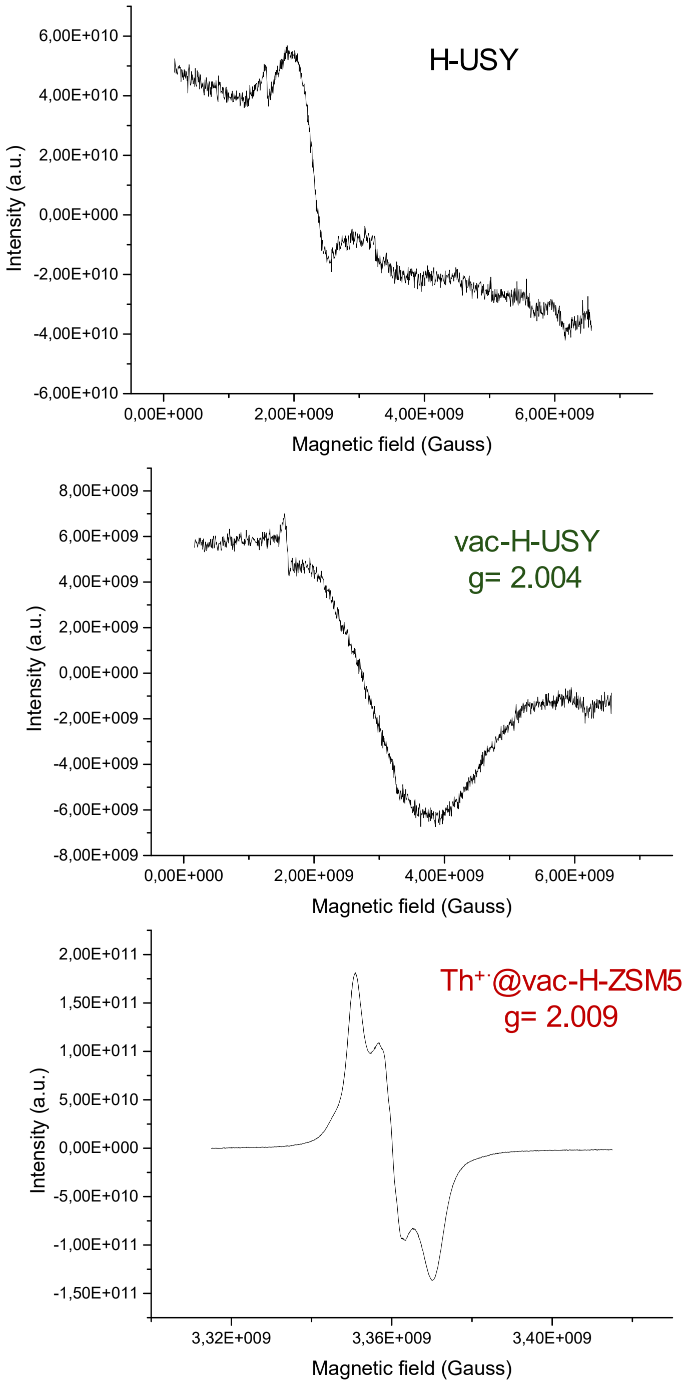

Figure S14. Electronic paramagnetic resonance (EPR) measurements of the different zeolites.

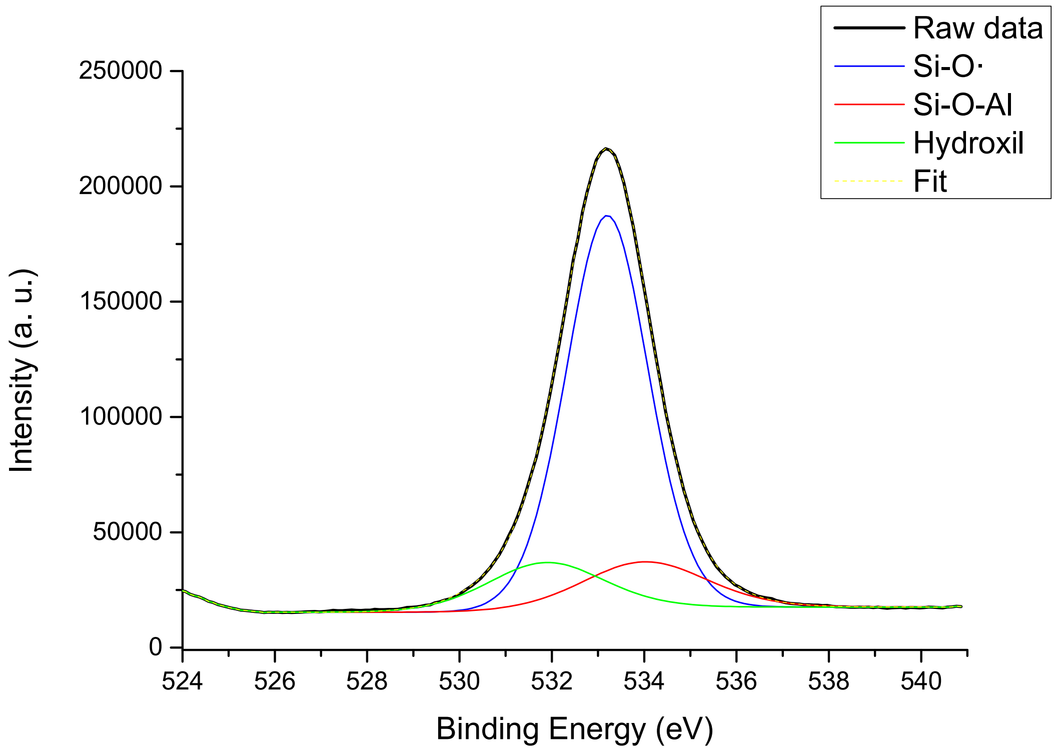

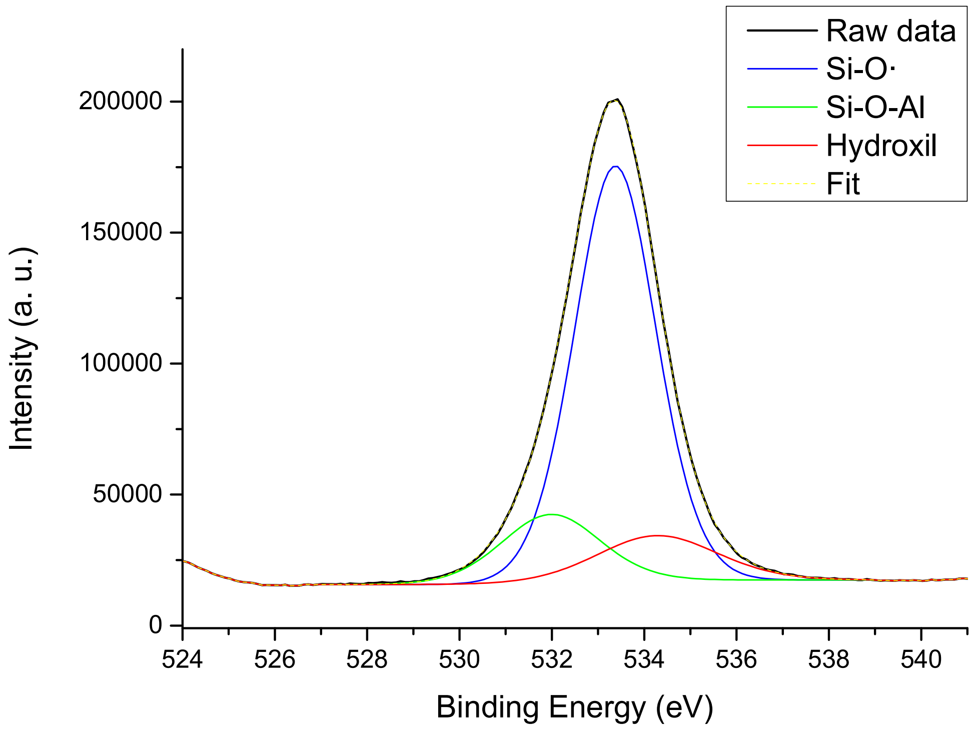

Figure S15. O 1*s* XPS measurements. The O 1s spectrum of H-USY (top) and vac H-USY (bottom) exhibited three peaks at 531.9, 533.2, and 534.0 eV, which are attributed to the O atom in NBOHC sites, the Si–O–Al network, and the hydroxyl group (also can be attributed to adsorbed water).

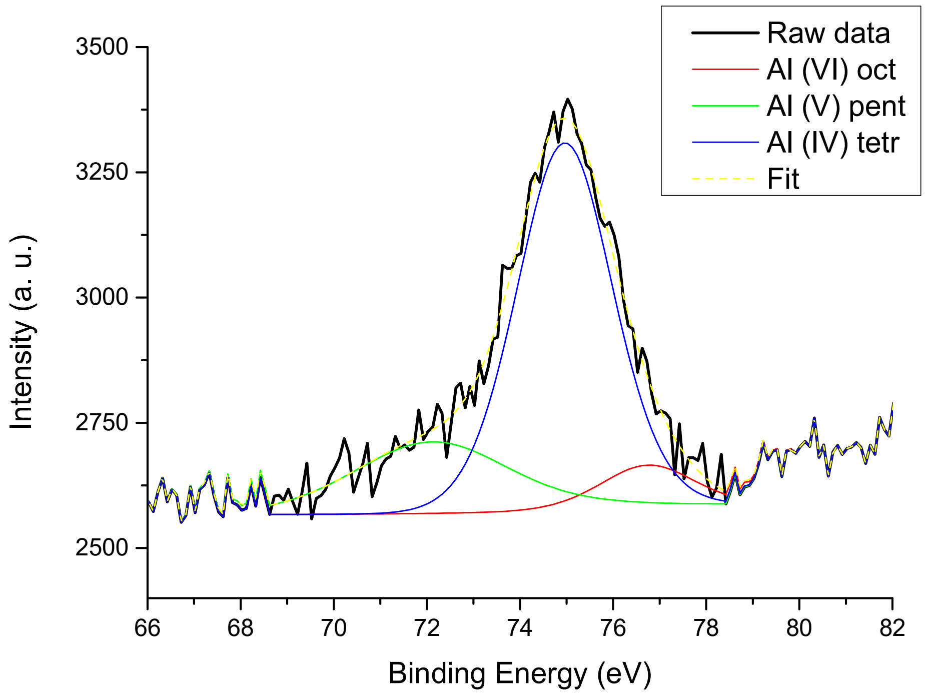

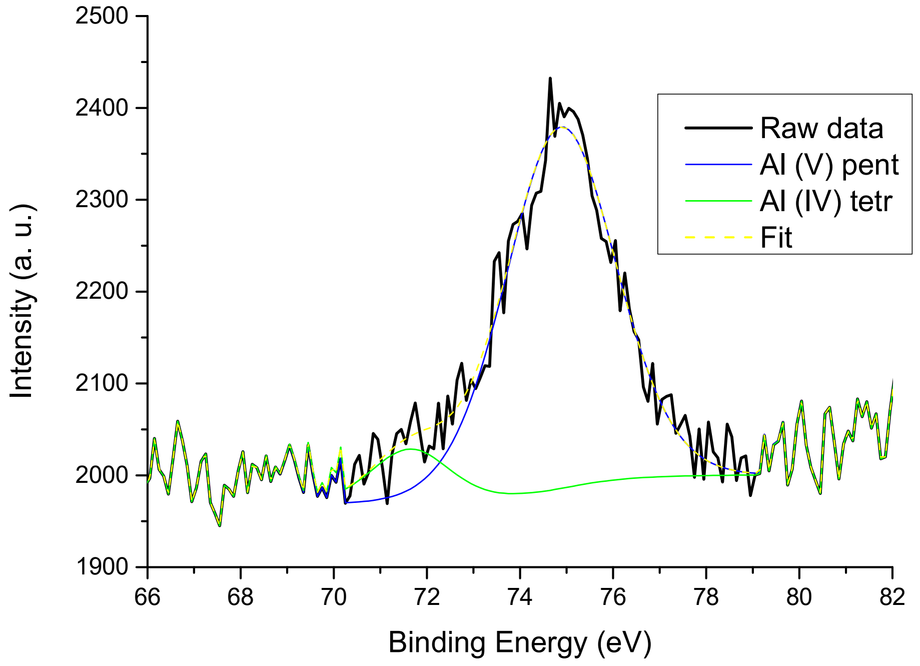

Figure S16. Al 2$p_{3/2}$ XPS analysis for bare H-USY (top) and vac-H-USY (bottom) zeolites.

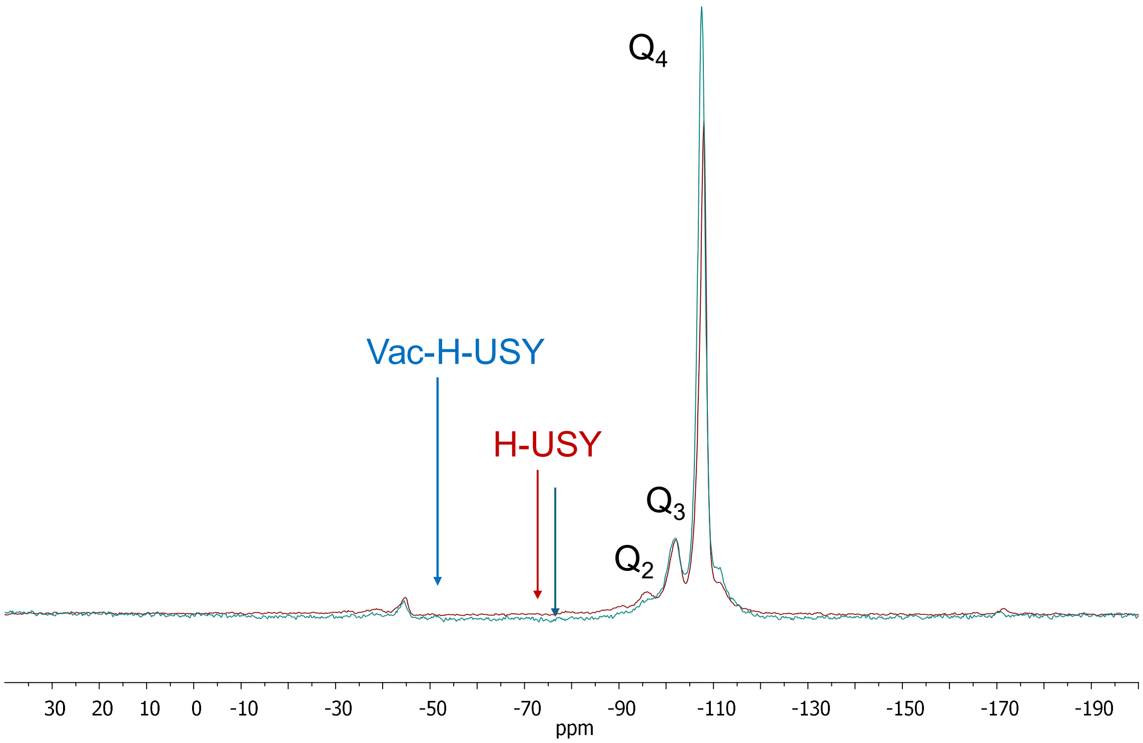

Figure S17. ^{29}Si MAS-SS-NMR measurements for the pristine H-USY zeolite (red line) and the vac-H-USY zeolite (blue line). A clear increase in the signal for Q_4 sites [Si-(OSi)$_4$] can be observed for the vac-H-USY zeolite.

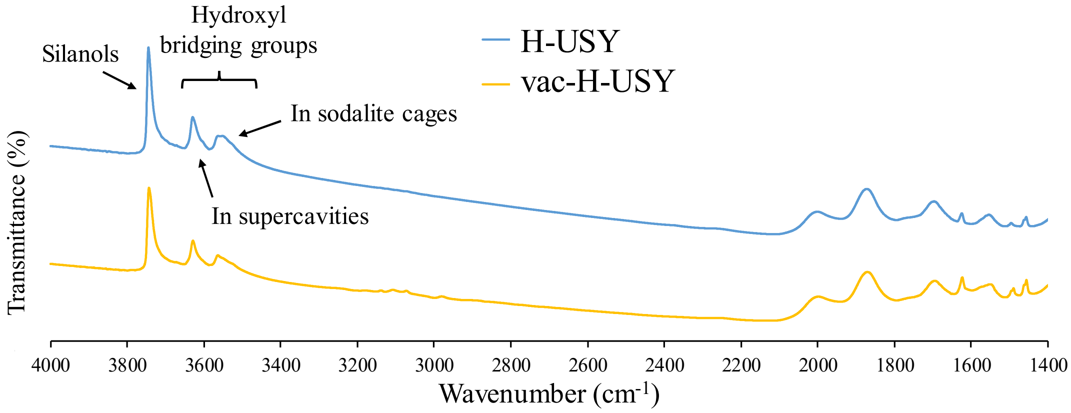

Figure S18. FT-IR spectra for the H-USY (top, blue line) and vac-H-USY zeolite (bottom, yellow line) after dehydration at 150 ºC under vacuum. The amount and type of silanols keep the same in both zeolites.

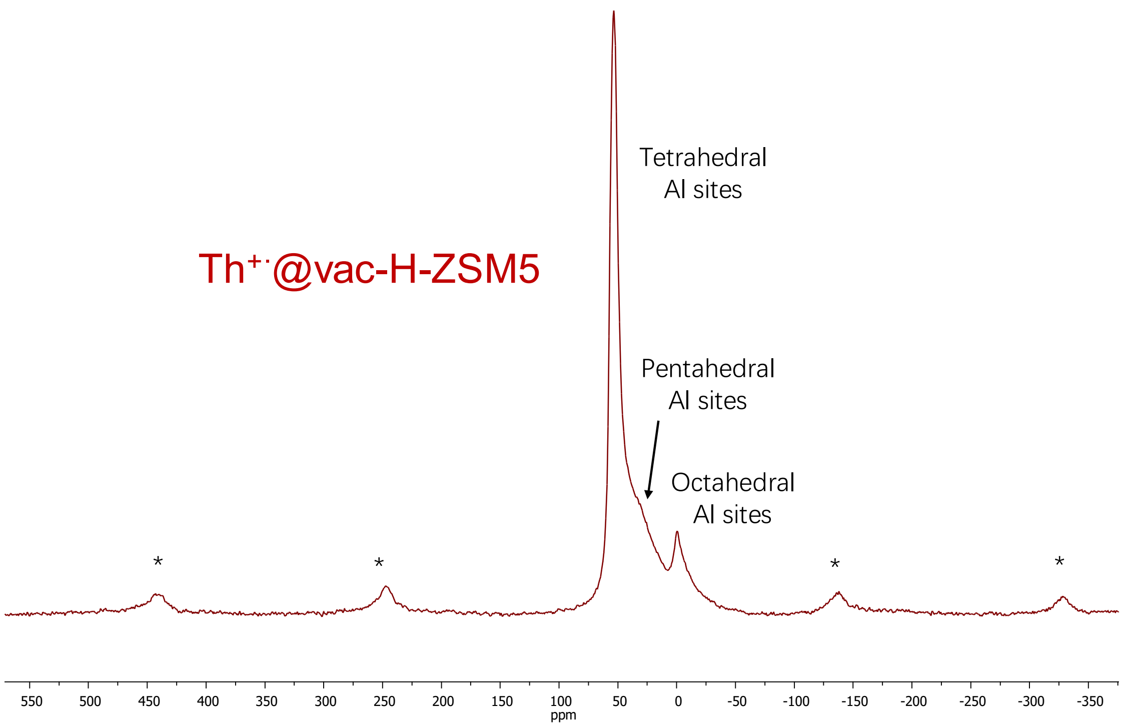

Figure S19. ^{27}Al MAS-SS-NMR spectrum for $Th^{+\cdot}$@vac-H-ZSM5. Asterisks (*) denote spinning signals.

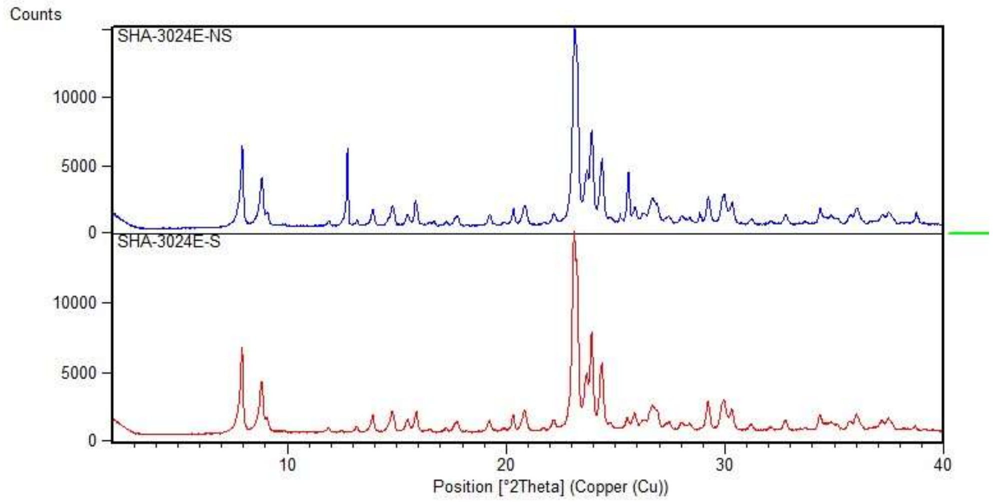

Figure S20. PXRD analysis of the $Th^{+\cdot}$@vac–H–ZSM5 before (top) and after Soxhlet washing (bottom). The disappearance of the peak at 12 degrees might be associated to a removal/migration of organic species from the zeolite channels.

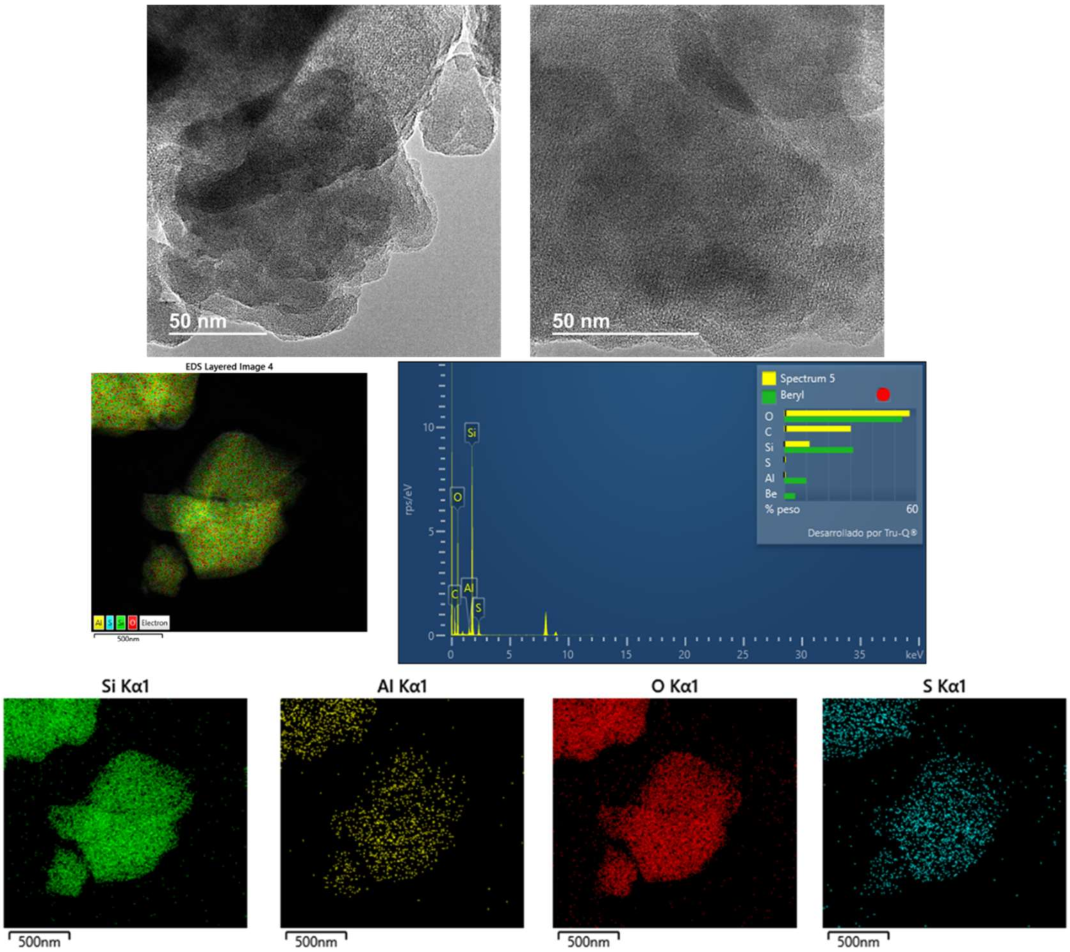

Figure S21. Representative high-resolution transmission electron microscopy (HR-TEM) images, with the electron diffraction X-ray (EDX) analysis and mapping of the vac–H–USY (no Soxhlet) with the Th still inside the zeolite.

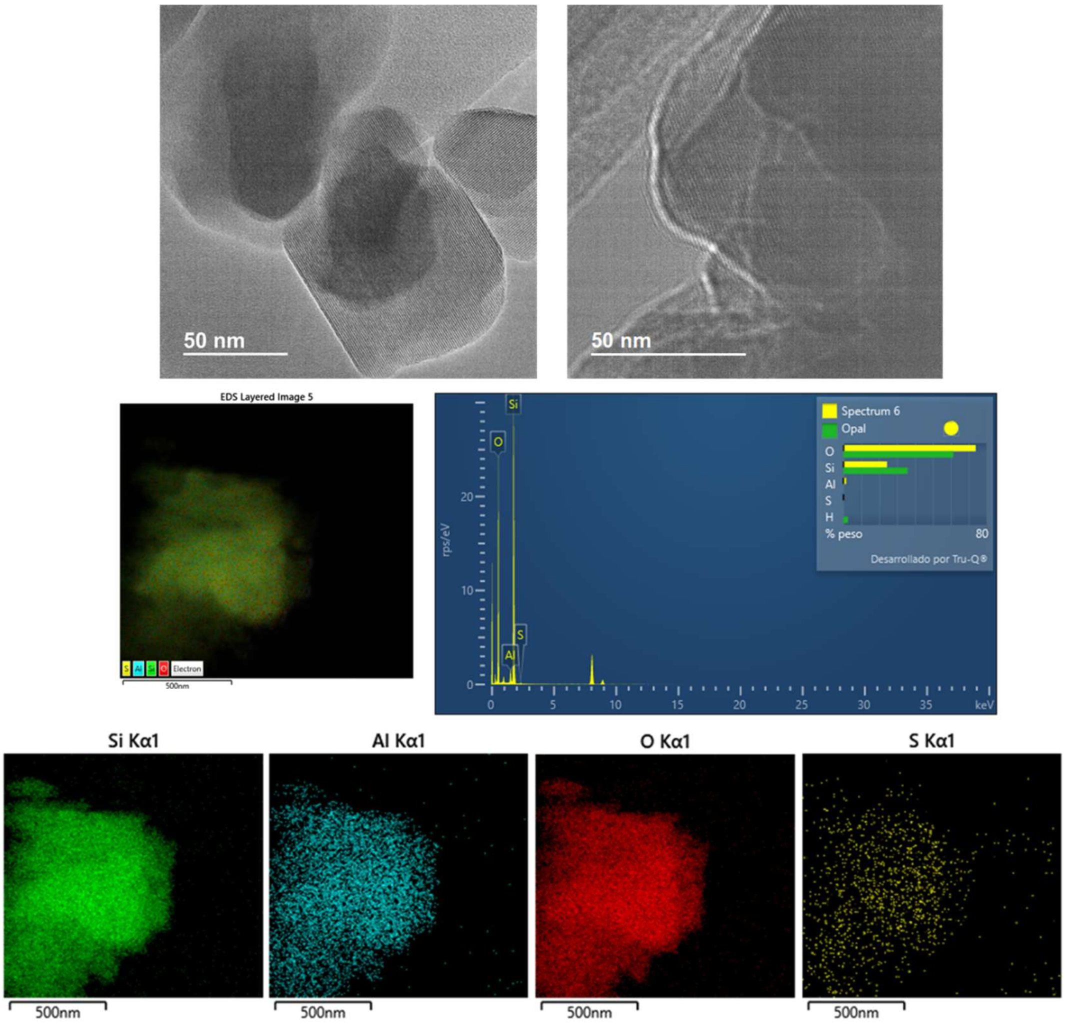

Figure S22. Representative high-resolution transmission electron microscopy (HR-TEM) images, with the electron diffraction X-ray (EDX) analysis and mapping of the $Th^{+\cdot}$@vac–H–ZSM5 zeolite.

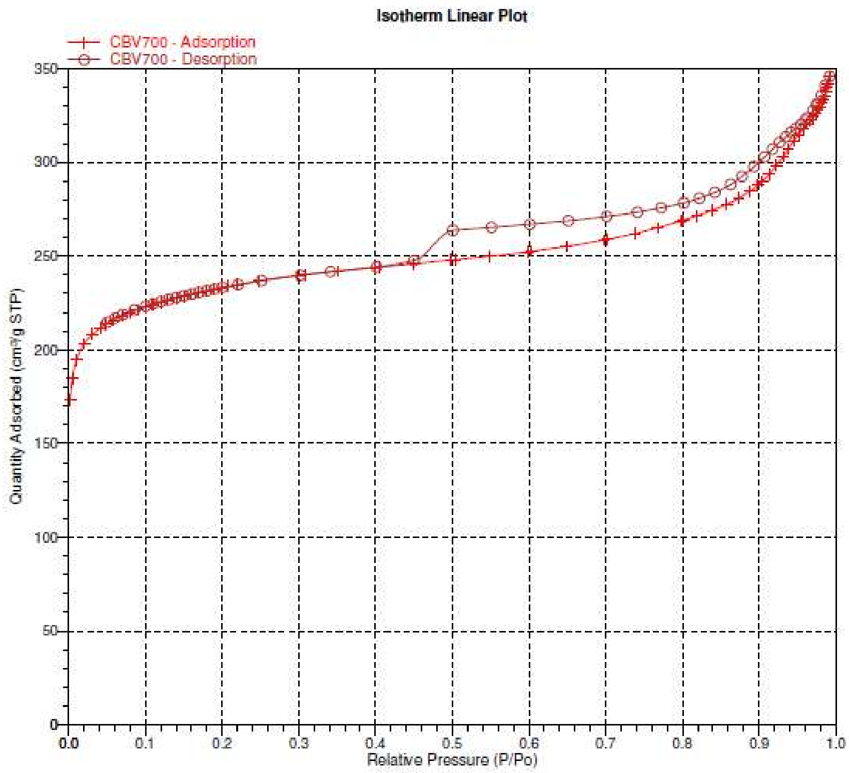

Figure S23. Isotherm plot for the vac-H-USY zeolite. The zeolite sample name corresponds to that given to the analytic service.

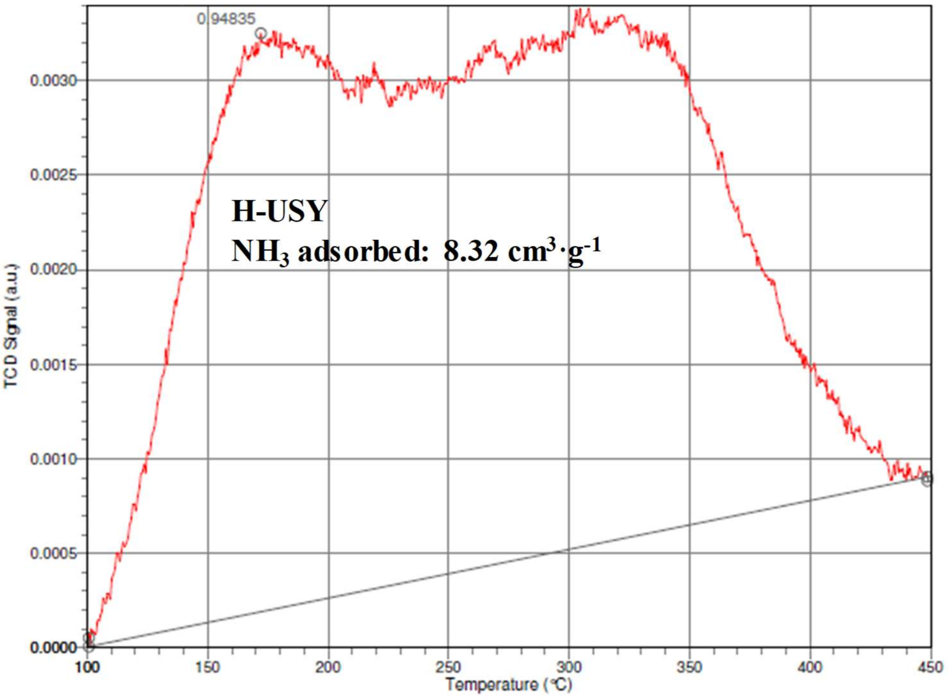

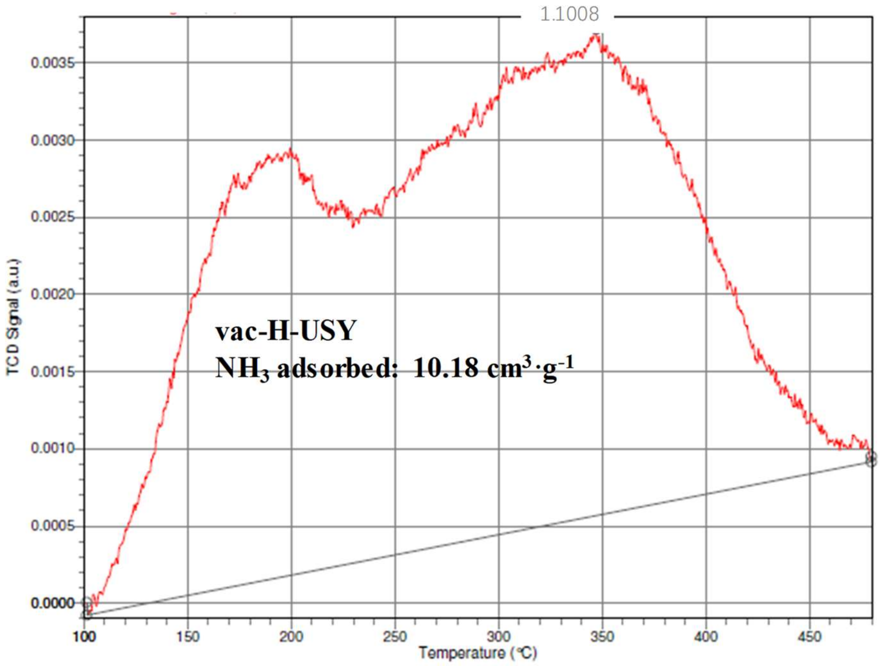

Figure S24. TPD of NH_3 in H-USY (top) and vac-H-USY zeolite (bottom) after dehydration at 300 ºC under vacuum. The amount of NH_3 adsorbed is indicated for both zeolites.

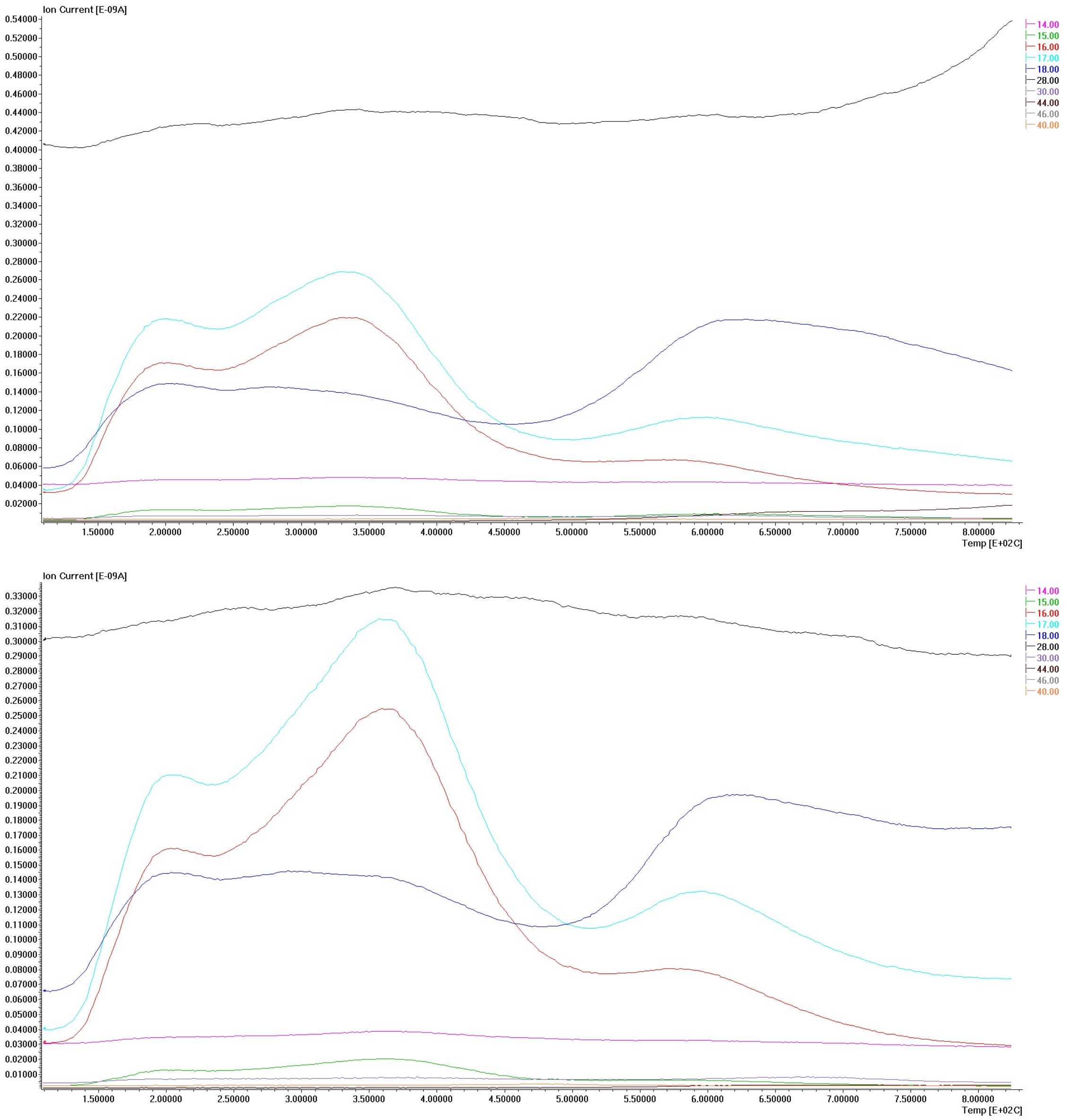

Figure S25. Thermogravimetric mass analysis during the TPD of NH_3 in H-USY (top) and vac-H-USY zeolite (bottom) after dehydration at 300 ºC under vacuum. The peak at 18 u.m.a is more presentative for NH_3 to avoid the interference of water at 17 u.m.a. The peak at 28 u.m.a. corresponds to N_2 as a reference.

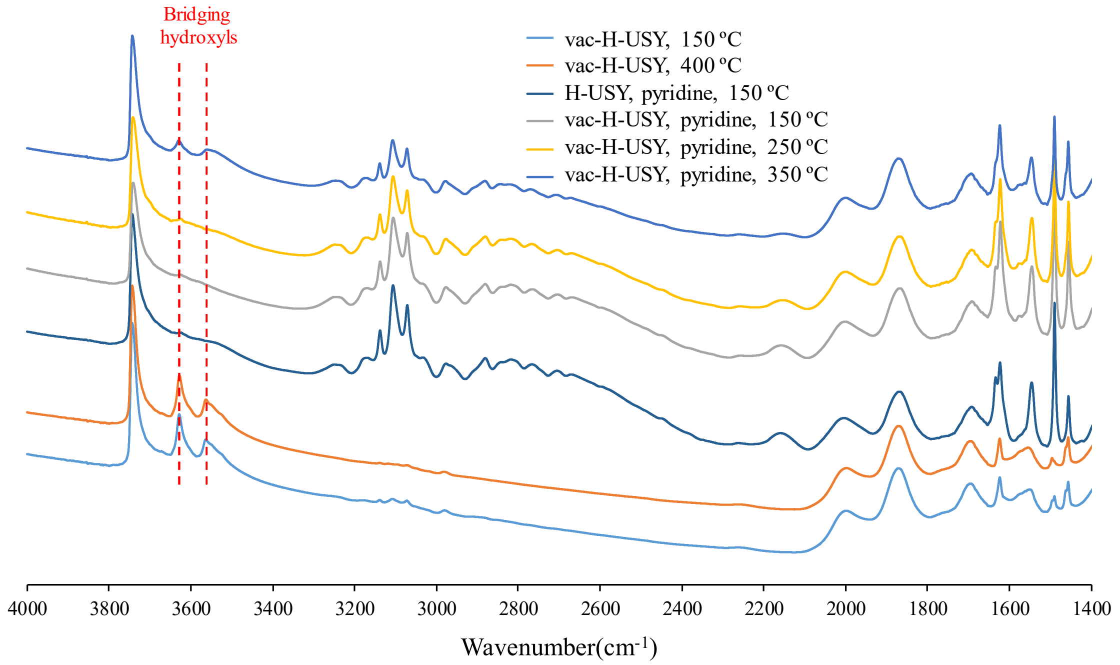

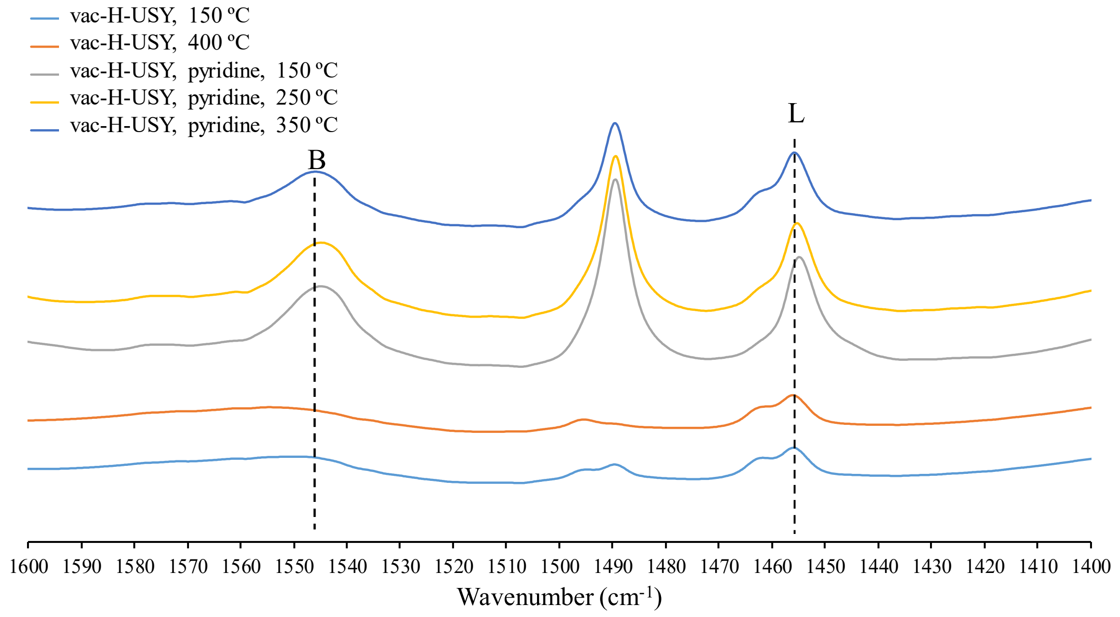

Figure S26. Top: Full FT-IR spectra for the vac-H-USY and H-USY zeolites with or without pyridine at different desorption temperatures (as indicated). Bottom: amplified diagnostic zone for pyridine.

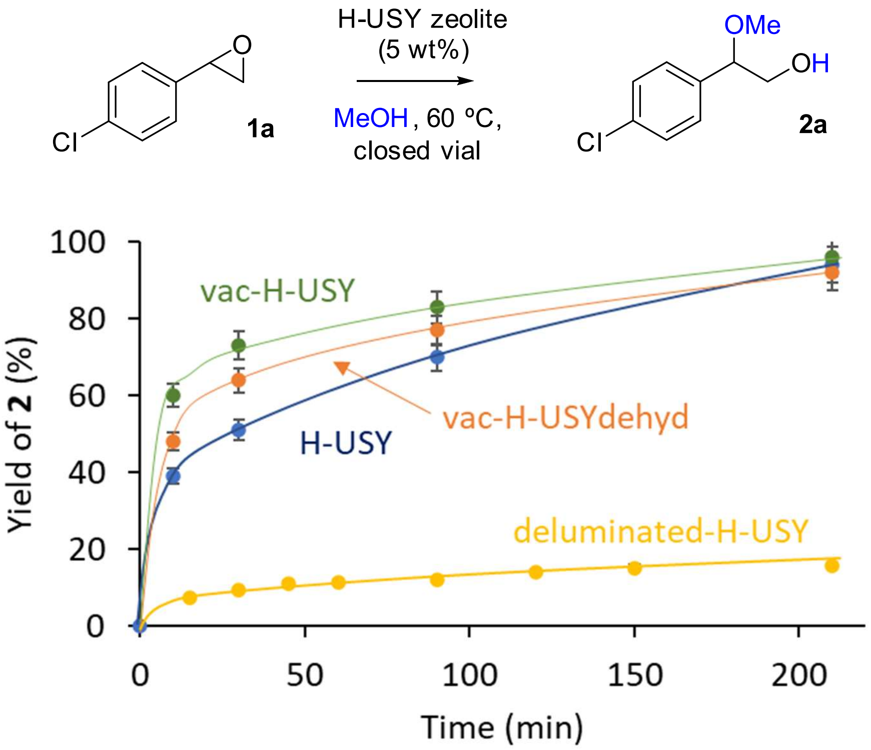

Figure S27. Kinetic plots for the hydroalkoxylation reaction of *p*–Cl–styryl epoxide **1a** or propylene epoxide **1b** (B) with MeOH (1M) in the presence of either H–USY, vac–H–USY, vac–H–USY–dehyd zeolite, and dealuminated-H-USY (5 wt%) at 60 ºC, to give product **2a**. No oligomeric products were found. GC results. Lines are a guide to the eye. Error bars account for a 5% uncertainty.

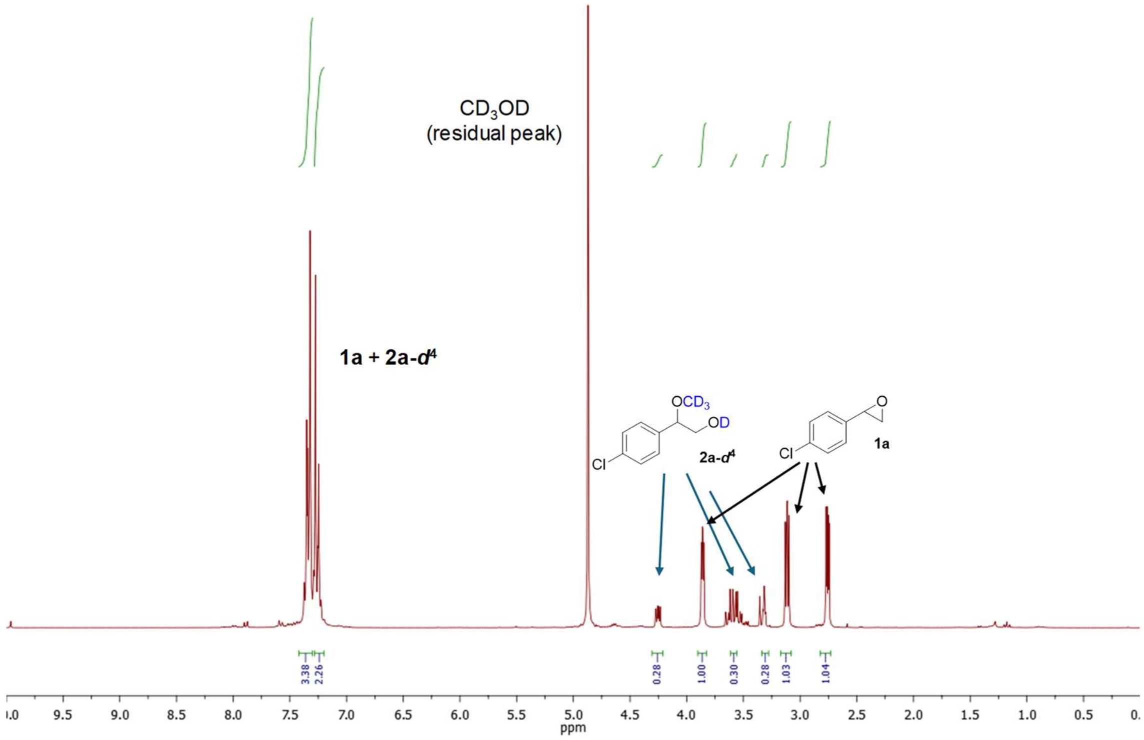

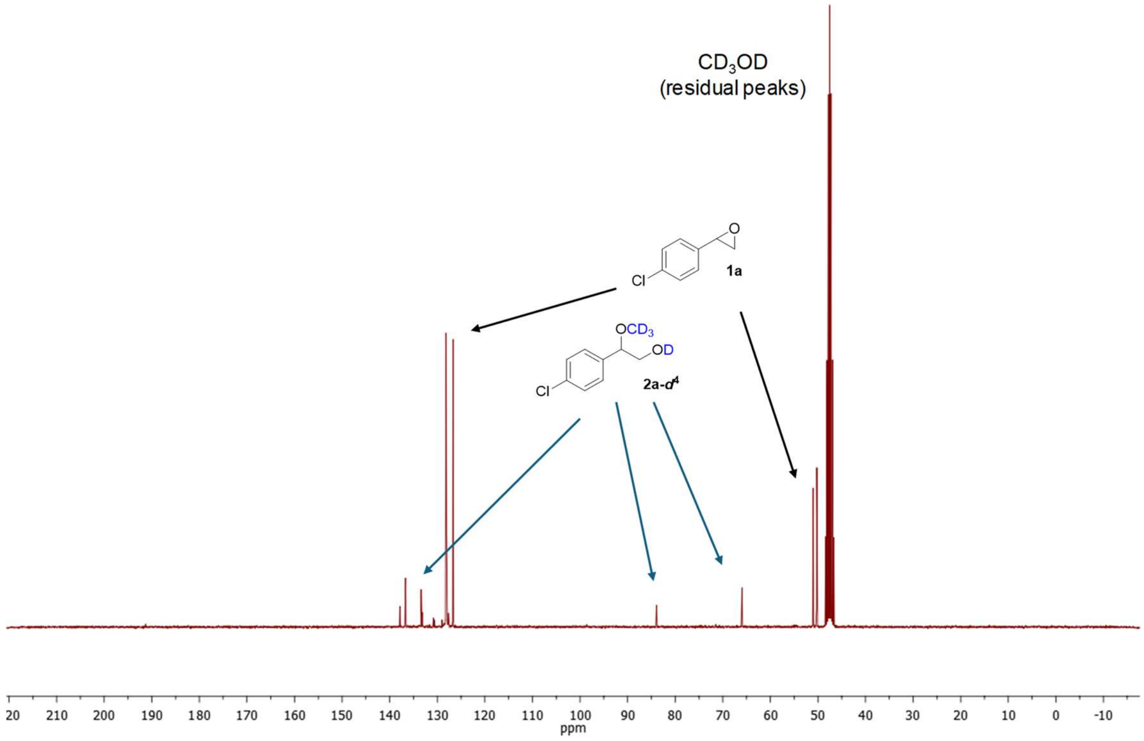

Figure S28. ^{1}H NMR (top) and ^{13}C NMR (bottom) for the hydroalkoxylation reaction of *p*-Cl-styryl epoxide **1a** with CD_3OD (1M) in the presence of vac-H-USY zeolite (5 wt%) at 60 ºC, after stopping at 25% conversion to give product **2a-*d*⁴**.

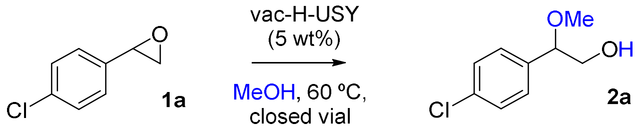

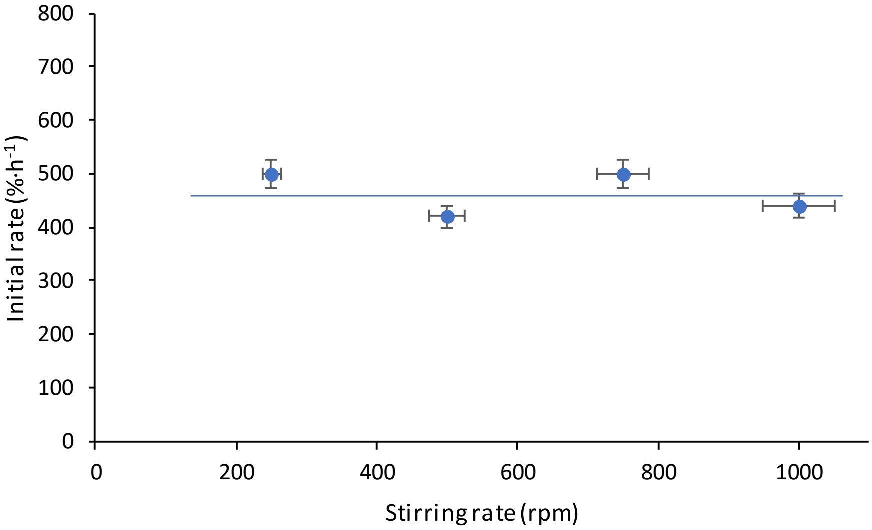

Figure S29. Initial reaction rate vs stirring rate for the hydroalkoxylation reaction of *p*-Cl-styryl epoxide **1a** with MeOH (1M) in the presence of vac-H-USY zeolite (5 wt%) at 60 ºC. Lines are a guide to the eye. Error bars account for a 5% uncertainty.

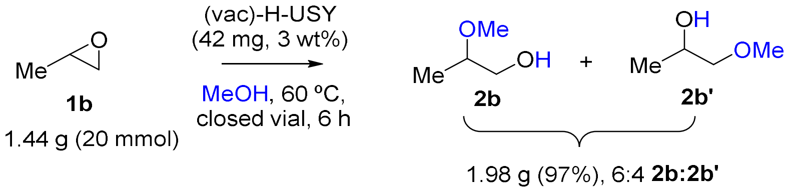

Figure S30. Gram-scale test for the hydroalkoxylation reaction of propylene epoxide **1b** with MeOH (1M) in the presence of vac-H-USY zeolite (3 wt%) at 60 ºC, to give products **2b** and **2b'**.

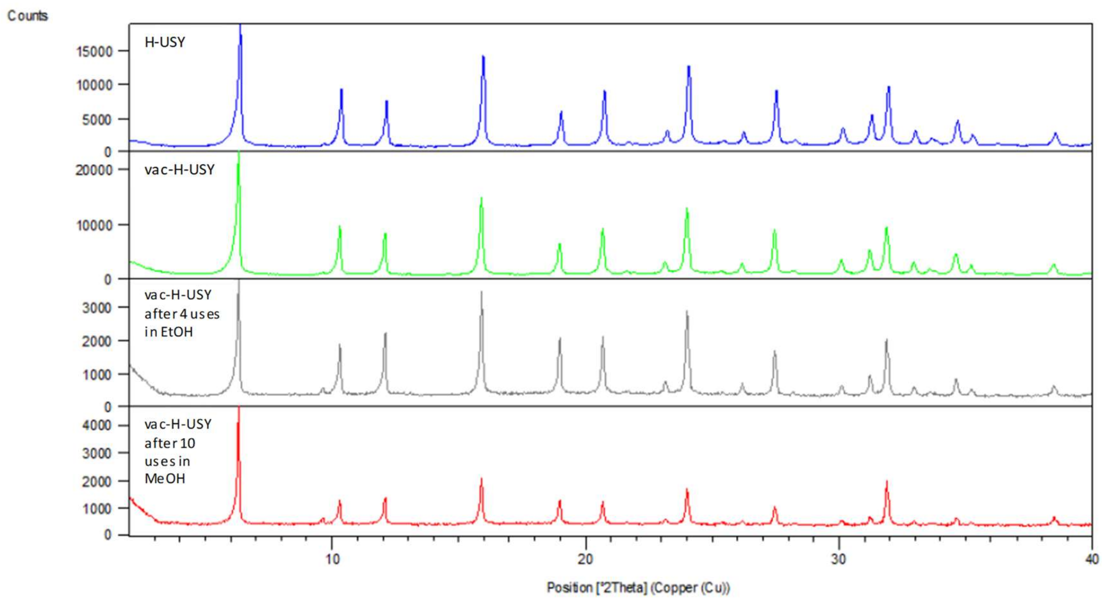

Figure S31. PXRD analysis of the bare H-USY, the functionalized H-USY (vac-H-USY), and the material recovered after 4 uses in EtOH and MeOH.

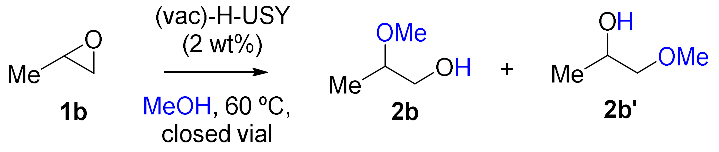

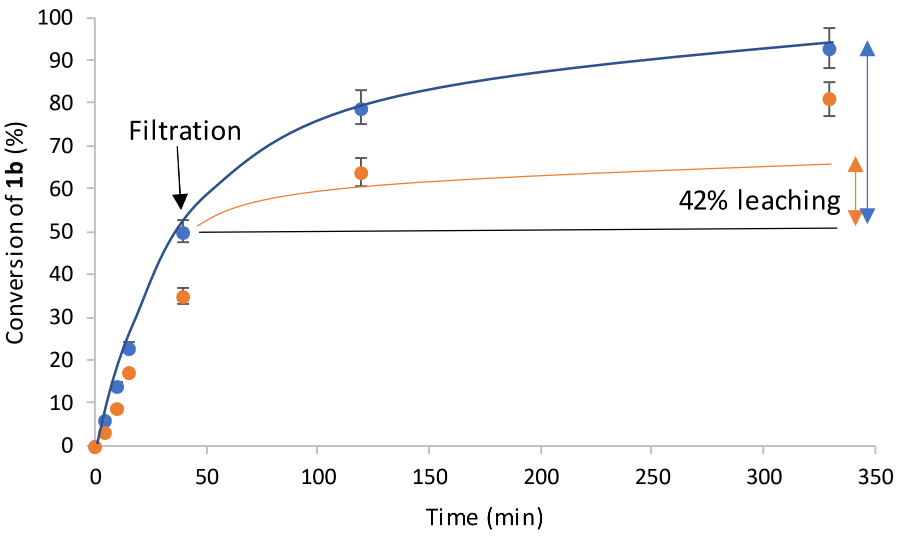

Figure S32. Leaching test for the hydroalkoxylation reaction of propylene epoxide **1b** with MeOH (1M) in the presence of vac-H-USY zeolite (2 wt%) at 60 ºC, to give products **2b** and **2b'**. Total conversion of **1b** is represented, the selectivity is 60:40 for **2b**:**2b'**. Error bars account for a 5% uncertainty.

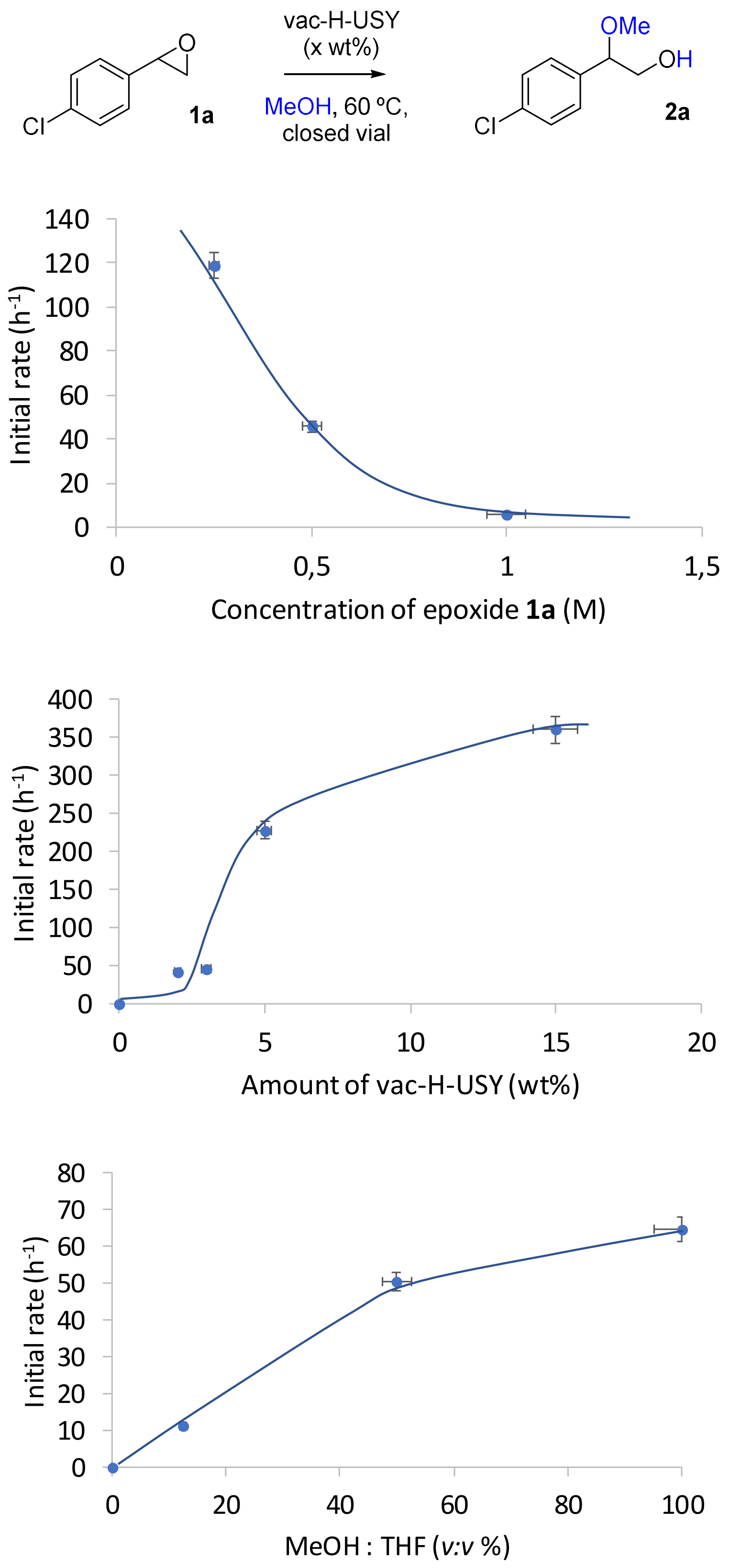

Figure S33. Initial reaction rate vs either the concentration of *p*-Cl-styryl epoxide **1a** (top), amount of vac-H-USY zeolite (middle) and relative amount of MeOH to THF (*v*:*v*, bottom), at 60 ºC. Lines are a guide to the eye. Error bars account for a 5% uncertainty.

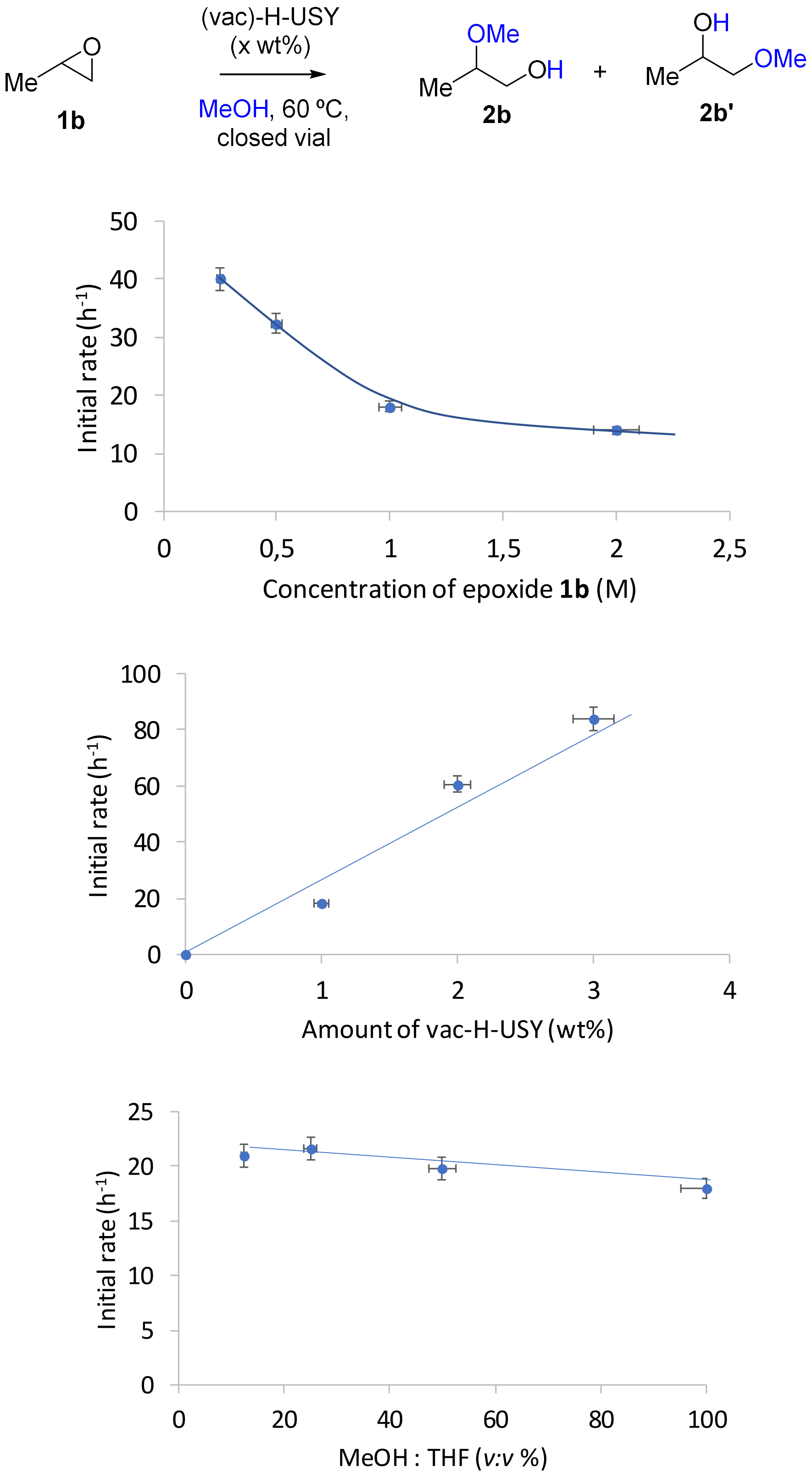

Figure S34. Initial reaction rate vs either the concentration of propylene epoxide **1b** (top), amount of vac-H-USY zeolite (middle) and relative amount of MeOH to THF (*v:v*, bottom), at 60 ºC. Lines are a guide to the eye. Error bars account for a 5% uncertainty.

Tables.

Table S1. Physicochemical properties of different solid acids employed in this work.

Entry	Solid Acid	Si/Al	BET surface area ($m^2 g^{-1}$)[a]	Total pore volume (cm^3/g^{-1})[a]	Particle or crystal size (mm)	Micropore volume (cm^3/g)[a]	Mesopore volume (cm^3/g)[a]
1	H-ZSM5	17.5	425	0.16[b]	0.5-2.0	0.016[b]	0.025[b]
2	H-USY	15	593	0.44	0.7-1.0	0.21	0.13
3	H-Beta	12.5	566	0.89	0.6	0.19	0.35
4	Al-MCM-41	15	853	0.72	0.4	N.D.	N.D.
5	ITQ-2	25	792	0.80	~ 0.5	0.04	0.48
**6**	**Vac-H-USY**	**15**	**758**	**0.52**	**0.7-1.0**	**0.17**	**0.14**

N.D. stands for "not determined". [a] BET surface area and total pore volume values were determined by nitrogen adsorption-desorption experiments. [b] Taken from the calcined H-ZSM5 sample.

Table S2. Weights obtained for the more representative zeolites in this study.

Entry	Zeolite	SiO_2/Al_2O_3	$m_{zeolita}$ (g)	$m_{zeolite}$ (g) after activation	$m_{thianthrene}$ (mg)
1	$Th^{+\cdot}$@vac-H-ZSM5	33	1.1010	1.0520	80.1
2	vac-H-USY	30	1.1267	1.1089	81.3
3	vac-H-USY-dehyd	30	1.1014	1.0307	82.9

Table S3. Elemental analysis results for the more representative zeolites in this study. The N content is residual from the tetra-alkyl ammonium directing agents used or the zeolite ammonium form, after calcination.

Entry	Zeolite	% N	% C	% H	% S	% Th
1	$Th^{+\cdot}$@vac-H-ZSM5	0.57	1.93	0.94	0.25	0.82
2	vac-H-USY	0.23	2.08	1.11	0.42	1.40
3	vac-H-USY-dehyd	0.30	1.77	1.08	0.22	0.73
4	vac-H-USY-dehyd (no Soxhlet)	0.30	13.11	2.78	2.78	9.11

Compound characterization.

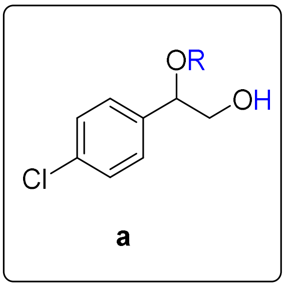

2a, R = Me. GC-MS (m/z, M^+ 186): 186, 169, 155 (100), 139, 125, 111, 91, 77, 51. ^1H NMR (401 MHz, CD_3OD): δ (ppm) 7.35-7.25 (m, 4H), 4.26 (dd, *J* = 7.6, 4.0 Hz, 1H), 3.63 (dd, *J* = 11.6, 7.6 Hz, 1H), 3.55 (dd, *J* = 11.6, 4.0 Hz, 1H), 3.32 (s, 3H). ^{13}C NMR (101 MHz, CD_3OD): δ (ppm) 138.7 (C), 134.6 (C), 129.7 (2xCH), 129.6 (2xCH), 85.4 (CH), 67.4 (CH_2), 56.3 (CH_3).

4a, R = Bn. GC-MS (m/z, M^+ 262) 264-262, 233-231, 139, 91 (100), 77, 65, 51. ^1H NMR (401 MHz, $CDCl_3$): δ (ppm) 7.39 – 7.28 (m, 9H), 4.62 (s, 1H), 4.52 (d, *J* = 11.2 Hz, 1H), 4.49 (dd, *J* = 8.0, 3.6 Hz, 1H), 4.33 (d, *J* = 11.2 Hz, 1H), 3.69 (dd, *J* = 11.6, 8.0 Hz, 1H), 3.59 (dd, *J* = 11.6, 3.6 Hz, 1H). ^{13}C NMR (101 MHz, $CDCl_3$): δ (ppm) 137.7 (C), 137.1 (C), 134.6 (C), 129.9 (2xCH), 129.0 (2xCH), 128.5 (2xCH), 128.1 (2xCH), 127.9 (CH), 81.7 (CH), 71.0 (CH_2), 67.2 (CH_2).

2b-3b were characterized by comparison with commercial samples after GC-MS analysis.

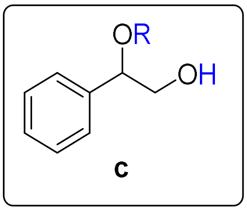

2c, R = Me. GC-MS (m/z, M^+ 152): 152, 135, 121 (100), 105, 91, 77, 65, 51. ^1H NMR (401 MHz, CD_3OD): δ (ppm) 7.43 – 7.15 (m, 5H), 4.26 (dd, *J* = 8.0, 4.0 Hz, 1H), 3.60 (dd, *J* = 12.0, 8.0 Hz, 1H), 3.54 (dd, *J* = 12.0, 4.0 Hz, 1H), 3.31 (s, 3H). ^{13}C NMR (101 MHz, CD_3OD): δ (ppm) 138.7 (C), 130.6 (2xCH), 129.2 (CH), 127.3 (2xCH), 86.3 (CH), 67.8 (CH_2), 56.3 (CH_3).

3c, R = H. GC-MS (m/z, M^+ 138): 138, 121, 107(100), 91, 79 (100), 51.

4c, R = Bn. GC-MS (m/z, M^+ 228): 228, 197, 120, 105, 92, 77, 65, 51. ^1H NMR (401 MHz, $CDCl_3$): δ (ppm) 7.44 – 7.32 (m, 10H), 4.69 (s, 1H), 4.58 (m, 2H), 4.39 (s, 1H), 3.78 – 3.73 (m, 2H). ^{13}C NMR (101 MHz, $CDCl_3$): δ (ppm) 138.5 (C), 137.7 (C), 129.9 (2xCH), 129.1 (2xCH), 128.8 (CH), 128.6 (CH), 128.4 (CH), 128.1 (CH), 128.0 (CH), 127.2 (CH), 82.5 (CH), 70.9 (CH_2), 67.5 (CH_2).

5c, R = Et. GC-MS (m/z, M^+ 166): 166, 135(100), 121, 107, 91,79, 77, 65, 51. **5c''** 2 R = Et. GC-MS (m/z, M^+ 194): 193, 149, 131, 121, 103 (100), 91, 75, 65.

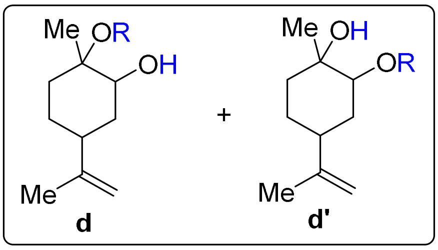

2d, R = Me. GC-MS (m/z, M^+ 184): 184, 169, 152, 134, 122, 108, 93, 85 (100), 72, 67, 55. **2d'**, R = Me. GC-MS (m/z, M^+ 184): 184, 169, 152, 134, 122, 109, 93, 85, 72, 67, 55.

3d, R = H. GC-MS (m/z, M^+ 170): 170, 152, 137, 123, 109, 108, 93, 82, 71 (100), 67, 58.

4d R = Bn. GC-MS (m/z, M^+ 260): 260, 169, 151, 136, 107, 91 (100), 71, 55. ^{1}H NMR (401 MHz, $CDCl_3$): δ (ppm) 7.46 – 7.32 (m, 5H), 4.86 – 4.85 (m, 2H), 4.79 (m, 1H), 4.44 (m, 1H), 3.82 (m, 1H), 2.44 – 2.33 (m, 1H), 1.98 – 1.56 (m, 6H), 1.84 (s, 3H), 1.21 (d, *J* = 6.4 Hz, 3H). **4d'**, R = Bn. GC-MS (m/z, M^+ 260): 260, 169, 161, 151, 139, 107, 91 (100), 71, 55. ^{1}H NMR (401 MHz, $CDCl_3$): δ (ppm) 7.46 – 7.32 (m, 5H), 4.86 – 4.85 (m, 2H), 4.79 (m, 1H), 4.44 (m, 1H), 3.59 (m, 1H), 2.44 – 2.33 (m, 1H), 1.98 – 1.56 (m, 6H), 1.78 (s, 3H), 1.21 (d, *J* = 6.4 Hz, 3H).

5d, R = Et. GC-MS (m/z, M^+ 198): 198, 183, 169, 152, 108, 99 (100), 86, 71, 58. **5d'**, R = Et. GC-MS (m/z, M^+ 198): 198, 180, 165, 152, 140, 136, 125, 108, 97, 82, 71 (100), 67, 59.

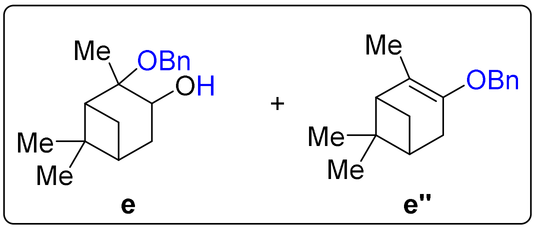

4e, R = Bn. GC-MS (m/z, M^+ 260): 260, 243, 152, 149, 137, 109, 91 (100), 79, 65, 55. **4e''**, R = Bn. GC-MS (m/z, M^+ 242): 242, 174, 151, 109, 91 (100), 81, 77, 55.

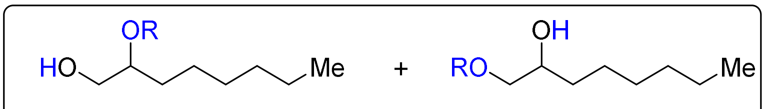

f + f'

2f, R = Me. GC-MS (m/z, M^+ 160): 159, 143, 129 (100), 97, 75, 69, 55.^{1}H NMR (401 MHz, CD_3OD): δ (ppm) 3.72 – 3.67 (m, 1H), 3.60 – 3.48 (m, 2H), 3.30 (s, 3H), 1.52 – 1.42 (m, 2H), 1.39 –1.32 (m, 8H), 0.94 – 0.91 (m, 3H). **2f'**, R = Me. GC-MS (m/z, M^+ 160): 159, 143, 129, 115, 97(100), 75, 69, 55.^{1}H NMR (401 MHz, CD_3OD): δ (ppm) 3.37 – 3.28 (m, 2H), 3.44 –3.21 (m, 4H), 1.52 –1.42 (m, 2H), 1.39 – 1.32 (m, 8H), 0.94 – 0.91 (m, 3H).

4f, R = Bn. GC-MS (m/z, M^+ 236): 236, 205, 107, 91 (100), 77, 65, 55. **4f'**, R = Bn. GC-MS (m/z, M^+ 236): 236, 205, 145, 122, 115, 107, 97, 91 (100), 79, 65, 55.

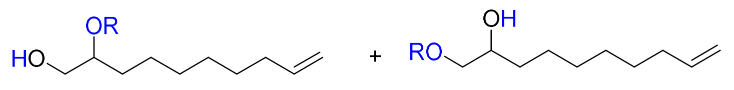

g + g'

2g, R = Me. GC-MS (m/z, M^+ 186): 186, 169, 155 (100), 123, 95, 81, 67, 55.^{1}H NMR (401 MHz, CD_3OD): δ (ppm) 5.89 – 5.75 (m, 1H), 5.03 – 4.87 (m, 2H), 3.73 – 3.68 (m, 1H), 3.61 – 3.49 (m, 2H) 2.93 (s, 3H), 2.11 – 2.05 (m, 2H), 1.57 – 1.35 (m, 10H). **2g'**, R = Me. GC-MS (m/z, M^+ 186): 186, 167, 141, 123, 95, 81 (100), 67, 55.^{1}H NMR (401 MHz, CD_3OD): δ (ppm) 5.89 – 5.75 (m, 1H), 5.03 – 4.87 (m, 2H), 3.38 – 3.31 (m, 2H), 3.27 – 3.21 (m, 1H) 2.93 (s, 3H), 2.11 – 2.05 (m, 2H), 1.57 – 1.35 (m, 10H).

4g, R = Bn. GC-MS (m/z, M^+ 262): 262, 231, 171, 153, 141, 123, 107, 91 (100), 81, 67, 55.

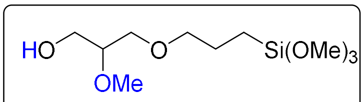

2h

2h, R = Me. GC-MS (m/z, M^+ 244): 244, 229, 215, 201, 186, 175 (100), 163, 147, 131, 124, 115, 105, 91, 82, 77, 67, 55. ^{1}H NMR (401 MHz, CD_3OD): δ (ppm) 3.73 – 3.72 (m, 1H), 3.69 – 3.63 (m, 1H), 3.60 – 3.49 (m, 5H), 3.55 (s, 9H), 3.37 (s, 3H), 1,88 – 1.84 (m, 2H), 0.89 – 0.85 (m, 2H).

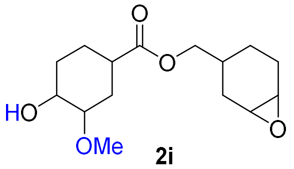

2i

2i, R = Me. GC-MS (m/z, M^+ 284):269, 252, 170, 159, 127, 111, 101 (100), 93, 79, 61, 55. ^{1}H NMR (401 MHz, CD_3OD): δ (ppm) 4.05 – 3.87 (m, 4H), 3.30 – 3.28 (m, 2H), 3.24 – 3.21 (m, 3H), 2.58 – 2.53 (m, 4H), 2.31 – 1.44 (m, 12H), 1.13 – 1.10 (m, 1H).

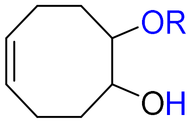

j

2j, R = Me. GC-MS (m/z, M^+ 156): 157, 142, 132, 124, 106, 96, 91, 80, 74, 67, 61(100), 54. ^{1}H NMR (401 MHz, CD_3OD): δ (ppm) 5.64 – 5.55 (m, 2H), 3.75 (td, *J* = 8.4, 3.6 Hz, 1H), 3.33 (s, 3H), 3.27 (td, *J* = 8.4, 3.6 Hz, 1H), 2.52 – 2.41 (m, 2H), 2.21 – 2.08 (m, 4H).

4j, R = Bn. GC-MS (m/z, M^+ 232): 232, 141, 123, 107, 95, 91 (100), 79, 67, 55.

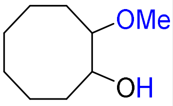

2k

2k. GC-MS (m/z, M^+ 158): 158, 144, 125, 109(100), 95, 82, 67, 55.

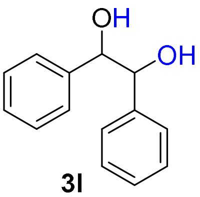

3l

3l. GC-MS (m/z, M^+ 214): 214, 196, 180, 179, 167, 165, 108 (100), 107, 89, 79, 77, 51.
